# Direct observational evidence of strong CO_2_ uptake in the Southern Ocean

**DOI:** 10.1126/sciadv.adn5781

**Published:** 2024-07-24

**Authors:** Yuanxu Dong, Dorothee C. E. Bakker, Thomas G. Bell, Mingxi Yang, Peter Landschützer, Judith Hauck, Christian Rödenbeck, Vassilis Kitidis, Seth M. Bushinsky, Peter S. Liss

**Affiliations:** ^1^ Centre for Ocean and Atmospheric Sciences, School of Environmental Sciences, University of East Anglia, Norwich, UK.; ^2^ Plymouth Marine Laboratory, Plymouth, UK.; ^3^ Flanders Marine Institute (VLIZ), InnovOcean Campus, Ostend, Belgium.; ^4^ Alfred-Wegener-Institut, Helmholtz-Zentrum für Polar- und Meeresforschung, Bremerhaven, Germany.; ^5^ Max Planck Institute for Biogeochemistry, Jena, Germany.; ^6^ School of Ocean and Earth Science and Technology, Department of Oceanography, University of Hawai'i at Mānoa, Honolulu, HI, USA.

## Abstract

The Southern Ocean is the primary region for the uptake of anthropogenic carbon dioxide (CO_2_) and is, therefore, crucial for Earth’s climate. However, the Southern Ocean CO_2_ flux estimates reveal substantial uncertainties and lack direct validation. Using seven independent and directly measured air-sea CO_2_ flux datasets, we identify a 25% stronger CO_2_ uptake in the Southern Ocean than shipboard dataset–based flux estimates. Accounting for upper ocean temperature gradients and insufficient temporal resolution of flux products can bridge this flux gap. The gas transfer velocity parameterization is not the main reason for the flux disagreement. The profiling float data–based flux products and biogeochemistry models considerably underestimate the observed CO_2_ uptake, which may be due to the lack of representation of small-scale high-flux events. Our study suggests that the Southern Ocean may take up more CO_2_ than previously recognized, and that temperature corrections should be considered, and a higher resolution is needed in data-based bulk flux estimates.

## Teaser

Strong CO_2_ sink observations in the Southern Ocean support flux estimates with temperature corrections and a higher resolution.

## INTRODUCTION

The Southern Ocean (south of 35°S) is a primary region for anthropogenic carbon dioxide (CO_2_) uptake, accounting for ~40% of the total ocean CO_2_ sink (*1*, *2*). Yet, it remains the most uncertain region with regard to CO_2_ flux estimates (*3*–*5*). This is essentially due to the sparsity of shipboard surface ocean CO_2_ fugacity (*f*CO_2w_) observations, especially during the austral winter (*6*–*8*). Since 2014, tens of profiling biogeochemical floats have been deployed in the Southern Ocean, and the data collected from these floats have addressed this wintertime data gap (*9*). Flux estimates based on the derived float *f*CO_2w_ data suggest a considerably weaker Southern Ocean CO_2_ sink in all seasons compared to the estimates based on the mainly shipboard dataset (fig. S1) (*10*, *11*). Global ocean biogeochemistry models (GOBMs) also simulate the CO_2_ flux (*12*). Although they largely agree with the shipboard *f*CO_2w_-based estimates on the annual mean flux (*3*), models have a large spread and indicate a weaker CO_2_ sink in austral summer and a stronger CO_2_ sink during winter in the Southern Ocean compared to the ship-based estimates (fig. S1).

In addition to uncertainties from sparse *f*CO_2w_ observations, upper ocean temperature gradients introduce another uncertainty. The *f*CO_2w_-based bulk flux estimate is sensitive to the temperature accuracy, and accounting for the ocean cool skin and the warm shipboard temperature bias results in a 15 to 30% increase in the Southern Ocean CO_2_ sink (*13*, *14*). The sampling alias (i.e., too-long sampling interval of the data) also leads to uncertainties in the estimate of mean CO_2_ flux (*15*, *16*). Intense but small-scale flux events may be important for the mean flux estimate in the Southern Ocean (*17*, *18*). Furthermore, the parameterization of gas transfer velocity (*K*_660_) remains a major source of uncertainty in air-sea CO_2_ flux estimates (*19*, *20*). Recent eddy covariance (EC) flux observations reveal substantial regional variations in the relationship of *K*_660_ to wind speed (*21*), but a uniform wind speed–dependent *K*_660_ is widely used to estimate the CO_2_ flux across different ocean regions.

Because of advancements in the EC technique, direct air-sea CO_2_ flux measurements on a largely autonomous basis (*22*) are now available to provide an independent constraint on the strength of the Southern Ocean CO_2_ sink. The EC technique measures CO_2_ flux directly (~10 km^2^, hourly resolution), which does not rely on any parameterizations of gas exchange and is thus not subject to subjective and often inconsistent choices of gas transfer velocity. Additionally, this micrometeorology method (i.e., EC) is unaffected by upper ocean temperature gradients and the sampling alias. Therefore, the direct flux measurements by EC provide an independent reference for any air-sea CO_2_ flux estimates. Over the period from 2019 to 2020, we collected extensive EC CO_2_ flux measurements during seven research cruises in the Southern Ocean (fig. S2). Here, we use these independent flux datasets to assess previous CO_2_ flux estimates in the Southern Ocean.

## RESULTS

### Mean air-sea CO_2_ fluxes

**Fig. 1.**
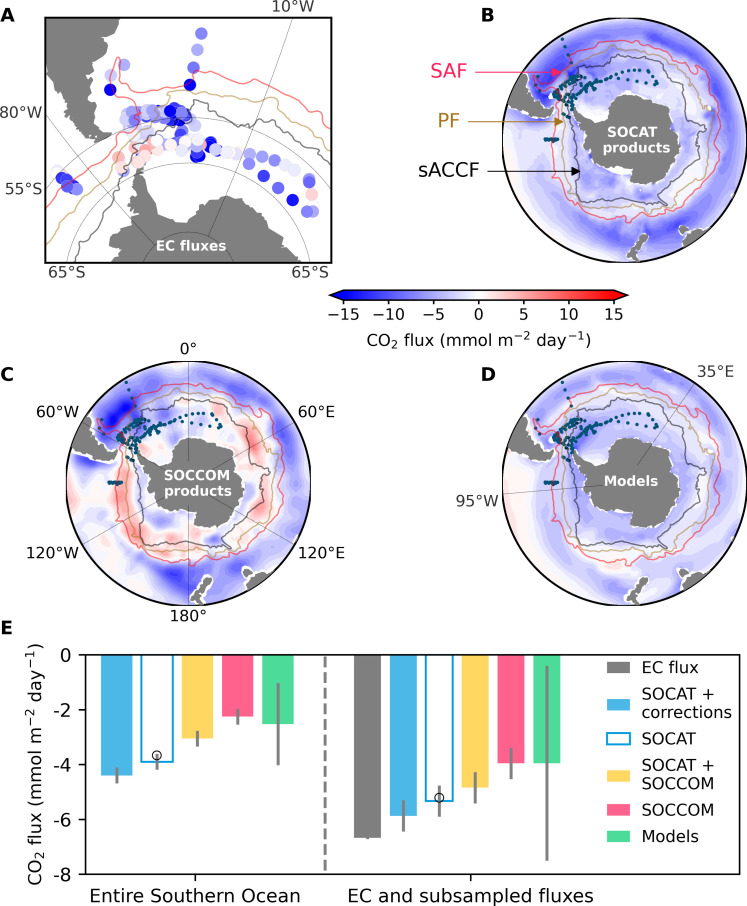
Austral summer (November to April) air-sea CO_2_ flux in the Southern Ocean. (**A**) Daily averaged EC CO_2_ flux measurements. Map of shipboard [(**B**), SOCAT]–, float [(**C**), SOCCOM]–, and GOBM [(**D**), models]–based CO_2_ flux estimates averaged over 2015 to 2020. (**E**) CO_2_ flux estimates for the entire Southern Ocean (left) and the mean of the hourly EC flux and subsampled flux estimates (right). Bars with different colors represent the EC flux measurements (black) and flux estimates from SOCAT-based flux products with (filled blue) and without (unfilled blue) temperature corrections, SOCAT plus SOCCOM–based products (yellow), SOCCOM-weighted products (red), and models (green). The same interpolation methods are used for the filled and unfilled blue bars. Open circles denote the two SOCAT-based flux products yielded through the same available interpolation methods as those for the SOCCOM-weighted products. Error bars indicate one standard deviation (SD) (see Materials and Methods). Fronts constructed from satellite altimetry data (*25*) are shown as red (SAF), brown (PF), and black lines (sACCF). Negative values indicate fluxes into the ocean.

We use ~2500 hours (~175 days) of high-quality EC air-sea CO_2_ flux measurements (*F*_EC_; Fig. 1A) to assess five CO_2_ flux estimates (see Materials and Methods) in the summertime (defined as November to April in this study) Southern Ocean:

1) *F*_SOCAT_corrections_: flux based on mainly shipboard *f*CO_2w_ observations in the Surface Ocean CO_2_ Atlas (SOCAT) dataset (*6*, *12*) with cool skin and warm bias temperature corrections (*13*)

2) *F*_SOCAT_: flux based on SOCAT dataset without cool skin and warm bias corrections

3) *F*_SOCCOM_: flux based on profiling float *f*CO_2w_ estimates from the Southern Ocean Carbon and Climate Observations and Modeling (SOCCOM) program south of 30°S (*9*) and named as SOCCOM-weighted product following a previous study (*10*)

4) *F*_SOCAT+SOCCOM_: flux based on the combined SOCAT and SOCCOM dataset south of 30°S (*10*)

5) *F*_models_: flux from GOBMs (*23*, *24*)

First, *F*_SOCCOM_ shows prevailing disagreements with *F*_SOCAT_, with the former on average 60% lower in magnitude than the latter (Fig. 1E) and their difference is most conspicuous in the frontal zone (Fig. 1, B and C). As expected, *F*_SOCAT+SOCCOM_ falls between *F*_SOCAT_ and *F*_SOCCOM_. In addition, *F*_models_ also indicates considerably lower CO_2_ uptake than *F*_SOCAT_, but their discrepancy is relatively uniform in space (Fig. 1, B and D) and the flux from different models has a large spread, as indicated by the large error bar in Fig. 1E. Furthermore, temperature corrections increase the SOCAT-based CO_2_ uptake by 13%. Consequently, our current knowledge of the strength of Southern Ocean CO_2_ sink in summer is *F*_SOCAT_corrections_ > *F*_SOCAT_ > *F*_SOCAT+SOCCOM_ > *F*_SOCCOM_ ≈ *F*_models_ in magnitude (Fig. 1E).

To assess these flux estimates using our EC data, we subsample the five CO_2_ flux products at the time and location of each hourly EC flux measurement. Most of the flux products originally have a 1° by 1°, monthly resolution (see Materials and Methods). The subsampled fluxes are expressed as *F*_SOCAT_corrections_sub_, *F*_SOCAT_sub_, *F*_SOCCOM_sub_, *F*_SOCAT+SOCCOM_sub_, and *F*_models_sub_. The EC flux suggests an on average 25% (1.4 mmol m^−2^ day^−1^) greater CO_2_ uptake than *F*_SOCAT_sub_, and a smaller (14%) difference with *F*_SOCAT_corrections_sub_ (Fig. 1E). *F*_SOCCOM_sub_ and *F*_models_sub_ (with large uncertainty) indicate a substantially weaker (~70%) CO_2_ uptake compared to the EC flux observations. It is worth noting that although the magnitude of the subsampled fluxes exceeds the corresponding mean fluxes for the entire Southern Ocean, the order of the different flux estimates is identical to that for the entire Southern Ocean (Fig. 1E). This suggests that while the observed ocean area is a relatively strong CO_2_ uptake region, the order of these five subsampled fluxes can effectively represent that of the entire Southern Ocean.

### Regional and temporal breakdown

The disagreement of the Southern Ocean CO_2_ flux among different estimates is not uniform in both space and time (Fig. 1 and fig. S1). Our extensive EC dataset, collected over 6 months during seven cruises and covering vast regions, allows for a comprehensive comparison with flux estimates across different regions and months. Notably, the SOCAT plus SOCCOM–based flux consistently falls between the SOCAT-based and SOCCOM-weighted flux estimates (fig. S1), and thus is excluded for the subsequent comparisons.

**Fig. 2.**
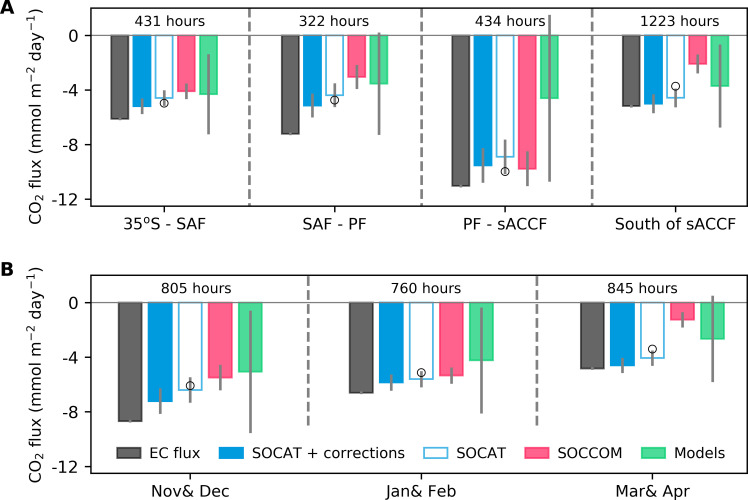
Regional and temporal breakdown of the EC CO_2_ flux measurements and subsampled flux estimates. In (**A** and **B**), the five bars with different colors represent the hourly EC flux measurements from the seven cruises (black), subsampled flux from SOCAT-based flux products with (filled blue) and without (unfilled blue) temperature corrections, SOCCOM-based flux products (red), and models. Open circles denote the two SOCAT-based flux products obtained using the same available interpolation methods as those for the SOCCOM-weighted products. Error bars reflecting one SD provide a measure of uncertainty (see Materials and Methods). Refer to the caption of Fig. 1 for the definition of the fronts SAF, PF, and sACCF. The number of hours of matched EC flux and subsampled flux is indicated above each subplot.

Previous research emphasizes the critical role of oceanographic fronts in driving discrepancies in different CO_2_ flux estimates (*10*, *11*). Here, we categorize the observed EC and subsampled fluxes into four distinct regions (*25*): between 35°S and the Subantarctic Front (SAF), between SAF and the Polar Front (PF), between PF and the southern Antarctic Circumpolar Current (sACCF), and south of sACCF (Fig. 1). Across all four regions, the EC flux shows consistently stronger CO_2_ uptake compared to all the subsampled flux estimates (Fig. 2A). The discrepancy value between *F*_EC_ and *F*_SOCAT _sub_ is relatively constant, and when accounting for the temperature corrections, the SOCAT-based flux estimate agrees better with the EC flux. *F*_SOCCOM_sub_ is substantially lower in magnitude than *F*_EC_, especially south of sACCF and in the area between SAF and PF. *F*_EC_ suggests a CO_2_ uptake approximately 2.5 times greater than *F*_SOCCOM_sub_ south of sACCF, while *F*_SOCAT_corrections_sub_ is very similar to the EC flux in this region. Given that most of our observations were south of the sACCF, the comparison in this region should be relatively robust. On a bimonthly timescale, the independent EC flux agrees reasonably well with *F*_SOCAT_corrections_sub_, while *F*_EC_ suggests consistently greater CO_2_ uptake compared to other subsampled flux estimates (Fig. 2B). *F*_SOCCOM_sub_ substantially underestimates the observed EC flux, particularly in March and April.

In summary, the reasonable agreement between the EC flux and *F*_SOCAT_corrections_sub_, alongside the substantial discrepancy between the EC flux and *F*_SOCCOM_sub_ as shown in Fig. 1E, aligns coherently with the regional and temporal breakdown (see also the latitudinal and longitudinal breakdown in fig. S3). Moreover, the difference between *F*_SOCAT_sub_ and *F*_EC_ is relatively consistent, while the discrepancy between *F*_SOCCOM_sub_ and *F*_EC_ is not uniform.

### Small-scale flux variability

**Fig. 3.**
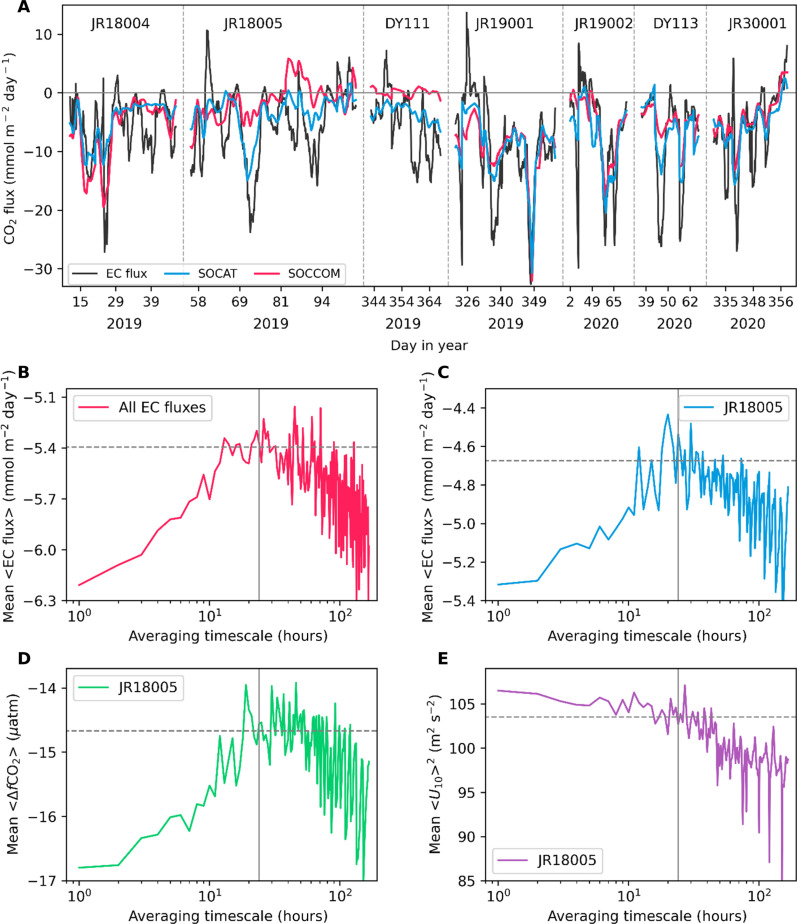
Flux time series with a daily running mean and the mean of the variables with different averaging timescales. (**A**) EC air-sea CO_2_ flux measurements from seven Southern Ocean cruises (black) and subsampled flux estimates from the average of two SOCAT-based flux products (blue) and two SOCCOM-weighted flux products (red) at the time and location of each hourly EC measurement, respectively. Note that *f*CO_2w_ observations from four (JR18004, JR18005, JR19001, and JR19002) of our seven cruises have been included in the SOCAT v2021 dataset. (**B**) Mean of the entire EC flux from seven cruises with different averaging timescales. (**C** to **E**) Mean of the EC flux (C), air-sea CO_2_ fugacity difference from the ship’s underway equilibrator *f*CO_2_ system [Δ*f*CO_2_, (D)], and the square of 10-m wind speed [*U*_10_, (E)] from cruise JR18005 with different averaging timescales. Note that all data in (C) to (E) are independent measurements. The solid-vertical line represents the 1-day timescale, and the dashed-horizontal line denotes the average of the mean flux with timescales between 16 and 32 hours.

The typical resolution of most CO_2_ flux products is 1° by 1° and monthly. The EC air-sea CO_2_ flux has a much higher temporal resolution of 1 hour and spatial resolution spanning ~10 km^2^. These high-frequency EC flux data provide valuable insights into small-scale flux variability. To reduce the random uncertainty, the hourly EC fluxes (fig. S4) are presented as a daily running mean in Fig. 3A (see Materials and Methods). The EC flux reveals mostly periods of ocean CO_2_ uptake with occasional short-lived outgassing events (Fig. 3A). The subsampled SOCAT-based and SOCCOM-weighted flux products closely track the daily EC flux variations (Fig. 3A) with a moderately to highly positive correlation coefficient (0.73 and 0.55, respectively). This suggests that the flux products based on the sparse *f*CO_2w_ data can reproduce the small-scale flux variability rather well. In addition, *F*_SOCCOM_sub_ indicates sustained CO_2_ outgassing during cruise JR18005 and a near-neutral flux environment during cruise DY111. Conversely, the direct EC flux observations suggest predominantly CO_2_ uptake during both cruises, supporting the SOCAT-based flux estimate. While the subsampled model flux can reflect the background flux signal, it does not capture most of the daily EC flux variations (*r* = 0.05; fig. S5), which is unsurprising because models have not been inherently designed to simulate small-scale flux processes.

None of the flux estimates can well capture the short-lived, high-flux events, which is potentially due to the coarse resolution of these flux products. However, these high-flux events may be important for the large-scale mean flux estimates. One of the SOCAT-based products [CarboScope, (*26*)] originally has a daily resolution. The subsampled daily CarboScope flux captures more high-flux events than the subsampled monthly aggregated flux (fig. S6), and the mean of the former is 13% higher in magnitude than the latter. Figure 3B suggests that the mean of the EC flux is sensitive to the averaging timescale with a ~15% flux decrease in magnitude from an hourly to a half-day scale. The mean EC flux has no obvious trend from a half day to 2 days, suggesting the steady state of the mean flux at this timescale domain. Beyond 2 days, the mean CO_2_ uptake shows an increasing trend with large fluctuations, which may be because the research vessels move across large regions and the natural spatial heterogeneity compromises the timescale sensitivity. This is supported by evidence that the mean of the subsampled monthly SOCAT-based flux products (i.e., *F*_SOCAT_sub_) is expected to have no trends, but shows an increasing trend in magnitude at timescales higher than 2 days (fig. S7). Thus, we do not consider the EC flux timescale analysis beyond 2-day timescales. The 15% EC flux decrease in magnitude from the hourly to the daily timescale can bridge the gap between *F*_EC_ and *F*_SOCAT_corrections_sub_ (14%). We use the observations from cruise JR18005, which has the fewest data gaps compared to other cruises (fig. S4), to test the possible reason for the mean flux sensitivity to the averaging timescale. The mean EC flux from JR18005 also shows a typical ~15% decrease in magnitude from an hourly to a half-day scale (Fig. 3C), which is primarily due to the decrease in the magnitude of Δ*f*CO_2_ instead of the wind speed (Fig. 3, D and E). This averaging timescale effect is essentially the sampling alias (i.e., sampling at insufficient spatial-temporal resolution). The change of the mean EC flux with the sampling interval (fig. S8) is similar to the change of the mean EC flux with the averaging timescale (Fig. 3).

### Gas transfer velocity

**Fig. 4.**
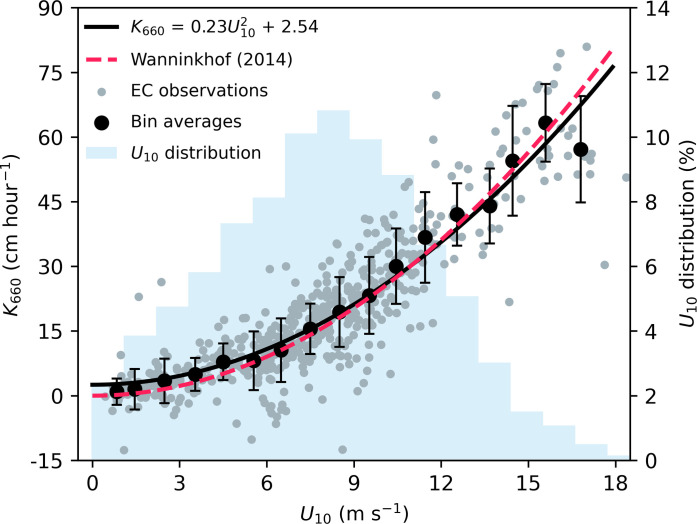
Gas transfer velocities (*K*_660_) derived from EC air-sea CO_2_ flux observations. Gray dots are hourly EC-derived *K*_660_ (553 hours), and black circles represent 1 m s^−1^ bin averages, with error bars indicating one SD. The black curve represents the least square fit using the bin averages (*R*^2^ = 0.78). The red dashed line corresponds to a *K*_660_ parameterization based on the global ^14^C inventory (*19*). Negative *K*_660_ values are due to uncertainties in EC fluxes and *f*CO_2_ observations. Light blue bars denote the frequency distribution of in situ wind speeds (*U*_10_) during our cruises.

The gas transfer velocity (*K*_660_) is a key parameter in both the *f*CO_2w_-based and model-based air-sea CO_2_ flux estimates (see Materials and Methods) and is often a source of inconsistency between estimates. Studies show that the uncertainty in the *K*_660_ parameterization dominates the overall uncertainty in global ocean CO_2_ uptake estimates [e.g., (*19*, *20*, *27*)]; thus, it behooves us well to test whether the discrepancies observed above can be linked to differences in the gas transfer. A common wind speed–dependent *K*_660_ constrained by the global bomb-^14^C inventory (*K*_660_14C_) (*19*) is used for CO_2_ flux estimates for the global ocean (*12*). However, a recent study shows that the *K*_660_–wind speed relationship has substantial regional variations, especially at low and high wind speeds (*21*). Our EC air-sea CO_2_ flux observations coupled with simultaneous *f*CO_2w_ observations made during the same cruise provide an opportunity to constrain *K*_660_ for the Southern Ocean environment from low to high wind speeds (see Materials and Methods). To minimize the impact of the cool skin effect on the EC-derived *K*_660_, we only use the data with |Δ*f*CO_2_| > 40 μatm for the parametrization. A total of 553 hours of *K*_660_ values are derived after quality control (Fig. 4), which is so far the most extensive ship-based high-quality *K*_660_ dataset in the Southern Ocean with consistent experimental setup and data processing (*22*).

The EC-derived *K*_660_ (*K*_660_EC_) on average agrees well with the *K*_660_14C_ at intermediate wind speeds (Fig. 4). However, *K*_660_EC_ is higher at low wind speeds and lower at high wind speeds compared to *K*_660_14C_, which is likely related to chemical enhancement and ocean waves (see Supplementary Text). Although this divergence is small (1 to 2 cm hour^−1^), it is notable in comparison to the global mean of gas transfer velocity (16.5 cm hour^−1^) (*28*). The results presented in Fig. 4 are based on the in situ wind speed measurements during our Southern Ocean cruises, while the *f*CO_2w_-based flux estimates typically rely on a reanalysis wind speed product (e.g., ERA5 and JRA55; see Materials and Methods). The mean difference between the square of the subsampled reanalysis wind speed product and the in situ wind speed is small (~3%). The use of subsampled wind speed from different wind products to parameterize *K*_660_ introduces slight changes in the coefficient (fig. S9). Nevertheless, the EC-based *K*_660_ consistently remains higher at low wind speeds and lower at high wind speeds compared to the ^14^C-based *K*_660_ parameterization (fig. S9). The re-calculation of *F*_SOCAT_sub_ using either our EC-based or the ^14^C-based *K*_660_–wind speed parameterization does not yield a substantial difference in the mean flux (~5%) and thus cannot explain the large difference in flux observed in Figs. 1 and 2. This is because intermediate wind speed (5 to 13 m s^−1^) conditions dominate our observations (Fig. 4), while the enhanced CO_2_ uptake at low wind speeds largely counteracts the dampened uptake at high wind speeds.

## DISCUSSION

The independent EC air-sea CO_2_ flux measurements suggest greater CO_2_ uptake than the subsampled SOCAT-based flux estimates (Figs. 1E and 2). The EC flux agrees better with the temperature-corrected SOCAT-based flux estimates (*13*). A previous study (*14*) reported a theoretically ~30% (0.35 Pg C year^−1^) increase in the SOCAT-based CO_2_ uptake in the Southern Ocean by considering the temperature effects (i.e., the ocean cool skin effect and the potential warm bias induced by the ship’s engine heating). This figure is revised to ~15% (0.2 Pg C year^−1^) with an updated assessment of these two temperature effects (*13*). This study provides observational evidence emphasizing the need to take these two temperature effects into account in SOCAT-based bulk flux estimates. Additionally, recent aircraft measurements in the south of 45°S also indicate a stronger ocean CO_2_ sink signal compared to *F*_SOCAT_, in agreement with this study (fig. S10) (*29*). Furthermore, the uncorrected SOCAT-based air-sea CO_2_ flux products (*12*), incorporating a riverine flux adjustment (*30*), yield a cumulative anthropogenic CO_2_ uptake of 47.9 Pg C for the decades 1994 to 2014. This is smaller than the anthropogenic CO_2_ uptake of 56.6 Pg C indicated by the interior ocean carbon inventory over the same period (*31*). The temperature corrections (*13*) increase the SOCAT-based CO_2_ uptake by 11.2 Pg C, bridging the gap between SOCAT-based and interior ocean inventory–based CO_2_ sink estimates, and resulting in near-zero non–steady-state natural carbon flux over these two decades. It is worth noting that the argument for considering the temperature corrections is from the comparison between *F*_EC_ and *F*_SOCAT_, but stronger evidence should be based on direct comparisons between *F*_EC_ and the bulk flux calculated by the simultaneously measured *f*CO_2_. However, our data collected by the research vessel is frequently calibrated and thus is free from the warm bias issue, and the impact of the cool skin correction on the in situ bulk flux is relatively small (−0.13 mmol m^−2^ day^−1^) compared to the large background flux in the regions with *f*CO_2_ observations (−5.5 mmol m^−2^ day^−1^). The in situ bulk flux shows good agreement with the EC flux (fig. S4B). A large fraction of the SOCAT data was collected by volunteer ships and lacked temperature calibration. Thus, both the warm bias and the cool skin effect have impacts on *F*_SOCAT_. Dedicated experiments with simultaneous EC and *f*CO_2_ observations at regions with |Δ*f*CO_2_| close to zero will be required to further confirm the cool skin flux correction.

The remaining difference between *F*_EC_ and *F*_SOCAT_corrections_sub_ can be explained by the insufficient temporal resolution of the SOCAT-based flux products (sampling alias). The high-flux events lasting less than a day are important for the mean flux estimate. Averaging over a too-long timescale or sampling over a too-large interval will dampen this high-flux effect and result in an underestimate of the mean CO_2_ flux (Fig. 3B). This sampling effect is mainly driven by the Δ*f*CO_2_ in our datasets (Fig. 3D), suggesting the need for high-resolution Δ*f*CO_2_ observations (i.e., hourly) and reconstruction in the Southern Ocean [fig. S6; (*15*–*18*)].

Relative to our independent EC flux data, the subsampled SOCCOM-weighted flux substantially underestimates the ocean CO_2_ uptake (Fig. 2). Particularly, a continuous CO_2_ outgassing period indicated by *F*_SOCCOM_sub_ is not supported by the EC flux observations, which suggest CO_2_ uptake (Fig. 3). The CO_2_ outgassing signal from *F*_SOCCOM_ is also not corroborated by the Southern Ocean aircraft campaigns (*29*). Moreover, the disagreement between *F*_SOCCOM_ and *F*_SOCAT_ not only is evident in winter but also prevails in summer (Fig. 1), the season when SOCAT contains more *f*CO_2w_ data than SOCCOM (fig. S11). Therefore, the disagreement between *F*_SOCCOM_ and *F*_SOCAT_ cannot be simply attributed to the sparsity in *f*CO_2w_ observations. Possible explanations for the mismatch include that SOCCOM *f*CO_2w_ values are not direct measurements but are derived from pH observations and total alkalinity estimates (*32*). Thus, the SOCCOM *f*CO_2w_ estimates have much larger theoretical uncertainties (±11 μatm) (*32*) compared to those of shipboard *f*CO_2w_ measurements (±2 to 5 μatm) (*6*). A positive bias may exist in these float *f*CO_2w_ estimates (+2 to +6 μatm) (*11*, *32*–*35*). It is also possible that *f*CO_2w_ mapping methods extrapolate local biased signals to the wider Southern Ocean. Correcting for an on average +4 μatm bias reduces the mean flux difference between *F*_SOCCOM_sub_ and *F*_EC_ already by half. Furthermore, the sampling alias may also be partially responsible for the underestimation of the SOCCOM-weighted flux given that the SOCCOM floats operate at a ~10-day sampling frequency (*9*). A recent study found that subsampling an hourly flux dataset in the Southern Ocean with a 10-day frequency results in a 23% positive bias (more outgassing/less uptake) in the mean flux (*16*). Another study indicates ±50% uncertainty in the mean flux with a 10-day sampling period, while the uncertainty is only 5% at a daily sampling frequency (*15*). Thus, we advocate against the use of the SOCCOM-weighted flux reconstruction, which was intended as an idealized experiment (*10*).

The ensemble mean of the eight process models considerably underestimates the observed EC air-sea CO_2_ flux (Figs. 1 and 2), and the subsampled fluxes from eight individual models have a large spread and different agreement with the EC flux (fig. S12). This is likely due to the models’ inadequate representation of biological processes in the summertime Southern Ocean (*3*) and insufficient resolution for capturing the small-scale processes (*17*). Notably, *f*CO_2_ observation–based CO_2_ flux estimates suggest a relatively robust capacity to reproduce daily flux variabilities (Fig. 3), which may provide valuable insights for refining models.

The gas transfer velocities derived from our EC CO_2_ flux measurements provide a constraint for *K*_660_ from low to high wind speeds at a scale (several square kilometers, hourly) comparable to that of the gas exchange processes. The good agreement in the bulk fluxes between using our EC-based *K*_660_ and the global ^14^C-based *K*_660_ (*19*) implies that the flux discrepancies presented in this study are not mainly due to *K*_660_. Consequently, the primary challenge in the Southern Ocean CO_2_ flux estimate lies in *f*CO_2_, highlighting the critical importance of sustaining efforts in high-quality and high-resolution *f*CO_2w_ observations. However, this *f*CO_2w_ collection effort has drastically declined in recent years and the number of the annual datasets in SOCAT decreased by 35% from 2017 to 2021 (40% in the Southern Ocean) (*36*).

This study suggests that the Southern Ocean may absorb more CO_2_ than previously recognized. It provides observational evidence for applying the temperature corrections and considering the sufficient temporal resolution in the shipboard dataset–based bulk flux estimates. In addition, the float-based flux product and models substantially underestimate the observed CO_2_ uptake. Noting that our cruise data only cover some part of the Southern Ocean in summer, continued efforts toward high-quality EC flux and *f*CO_2w_ observations are essential to improve the estimate of air-sea CO_2_ fluxes. This may include an expansion of simultaneous EC flux and *f*CO_2w_ measurements to more ships, and possibly the further deployment of buoys and Sail drones, especially for measurements in low |Δ*f*CO_2_| region to test the cool skin effect and the winter season with high speed. Moreover, refined resolutions in the *f*CO_2w_ reconstruction and model simulation should be a focus of future work.

## MATERIALS AND METHODS

### Direct flux measurements by EC

EC fluxes are measured in the atmosphere and do not rely on *f*CO_2_ measurements and gas transfer velocity parameterization. The air-sea CO_2_ flux *F* is measured directly by the EC technique and is calculated using

$$F=\rho \bar{w\prime c\prime }$$ (1)

where ρ is the mean mole density of dry air (e.g., in mol m^−3^). The CO_2_ mixing ratio in dry air *c* [in parts per million (ppm) or μmol mol^−1^] is measured by a fast-response gas analyzer with a dryer, and the vertical wind velocity *w* (in m s^−1^) is measured by a sonic anemometer and corrected for the ship’s motion. The prime denotes the fluctuations from the mean, while the overbar indicates the time average during the flux calculation interval: 20 min in this study. The sign of the EC flux is determined by the net number of CO_2_ molecules invading into and evading from the surface ocean within a flux interval.

Seven research cruises (fig. S2) were conducted in the Southern Ocean on two UK ships in the austral summer of 2019 and 2020. Air-sea CO_2_ fluxes were measured using a state-of-the-art closed-path EC system (Picarro G2311-f on RRS *James Clark Ross*, LI-7200 on RRS *Discovery*) with a dryer to eliminate the impact of water vapor fluctuations on the CO_2_ flux measurements during all these cruises (*22*). The EC data have been processed and filtered to meet the stationarity requirement of the EC method (*22*). EC flux measurements in regions with sea ice and close to land (distance from land less than 30 km) were removed to avoid confounding the open ocean. In total, we obtained ~3300 hours of quality-controlled EC air-sea CO_2_ flux measurements, corresponding to 175 days (at least 4 hours required per day to ensure the representativeness), which is so far the largest ship-based EC CO_2_ flux dataset with consistent instrumental setup and data processing. The random uncertainty in the hourly EC flux (~2 mmol m^−2^ day^−1^) will be considerably reduced after averaging over *n* hours ( $2/\sqrt{n}$ ) (*22*). Detailed descriptions of these cruises and the EC system are given in the Supplementary Materials.

### Bulk air-sea CO_2_ flux and product subsampling

Air-sea CO_2_ flux can be indirectly estimated by the bulk equation

$$F=K_{660}{\left(\mathrm{Sc}/660\right)}^{-0.5}\;\left(\alpha_{\text{ss}}f{\text{CO}}_{2w}-\alpha_{s}{f\text{CO}}_{2a}\right)$$ (2)

where *K*_660_ (cm hour^−1^) is the normalized gas transfer velocity at a Schmidt number (*Sc*) of 660 (*37*). α*_ss_* and α*_s_* are the CO_2_ solubility (mol liter^−1^ atm^−1^) (*38*) in the subskin and skin layers in seawater, respectively (*39*). *f*CO_2a_ (*f*CO_2w_) is the atmospheric (seawater) CO_2_ fugacity (in μatm). The current *f*CO_2w_-based flux products generally neglect the cool skin correction by assuming that α_ss_ is equal to α_s_ and using the same seawater temperature to calculate both.

To estimate the global ocean CO_2_ flux with Eq. 2, interpolating the sparse *f*CO_2w_ measurements to the global ocean is a key step. Seven SOCAT (*6*) v2021 dataset–based *f*CO_2w_ products using seven interpolation methods (*26*, *40*–*45*) have been made available for the Global Carbon Budget 2021 (GCB2021) (*23*). Among the seven interpolation methods, two of them [MPI-SOMFFN (*44*) and CarboScope (*26*)] have also been used to interpolate the SOCCOM *f*CO_2w_ estimates and the SOCAT plus SOCCOM datasets in the Southern Ocean from 2015 to 2020 inclusive (*10*). The *f*CO_2w_ product is combined with a global wind speed product [e.g., ERA5, (*46*)], a sea surface temperature [e.g., OISST v2, (*47*)] and salinity product, and a global *f*CO_2a_ product (NOAA Marine Boundary Layer dry air mixing ratio of atmospheric CO_2_ and corrected for water vapor pressure) to generate the CO_2_ flux product. We subsample CO_2_ fluxes from the seven SOCAT-based flux products (*F*_SOCAT_sub_) according to the time and location of the hourly EC observations. Then, we subsample the cool skin effect and warm bias flux corrections at a 1° by 1°, monthly resolution (*13*) and apply these flux corrections to *F*_SOCAT_sub_ to produce the temperature-corrected CO_2_ flux subsamples (*F*_SOCAT_corrections_sub_). The cool skin effect is simulated by a physical model (*48*), while the warm temperature bias is assessed by the buoy temperature dataset (*49*). The two SOCCOM-weighted (*F*_SOCCOM_sub_) and two SOCAT plus SOCCOM–based (*F*_SOCAT+SOCCOM_sub_) flux products are also subsampled at the time and location of the hourly EC observations. See Supplementary Text for how the SOCCOM data are being used in the interpolation process. The products are subsampled from their original resolutions (i.e., 2° latitude by 2.5° longitude, daily for CarboScope; 1° by 1°, monthly for the remaining products). The ensemble mean of the corresponding dataset-based flux products is used for analysis, and the SD of these seven individual SOCAT-based flux products is considered as the uncertainty for *F*_SOCAT_corrections_sub_, *F*_SOCAT_sub_, *F*_SOCCOM_sub_, and *F*_SOCAT+SOCCOM_sub_. The impact of the product resolution on the flux comparison is shown in fig. S6. Note that some of the *f*CO_2w_ data from our Southern Ocean cruises have been included in the SOCAT v2021 dataset (see Supplementary Text). In total, ~2500 hours of EC flux are matched with all the subsampled flux estimates and these matched data are used for comparison.

GOBMs constrain the air-sea CO_2_ flux by the transport of dissolved inorganic carbon from the surface into the ocean interior (*24*). We resampled CO_2_ flux from eight models (1° by 1°, monthly) used in GCB2021 (*23*) according to the time and location of the hourly EC observations. The ensemble mean of these eight subsampled model fluxes (*F*_models_sub_) is used for analysis, and their SD is assigned as the model flux uncertainty. Note that the model flux represents the anthropogenic CO_2_ sink, and the riverine flux should be adjusted to make it comparable with the EC flux observations and the *f*CO_2w_-based flux estimates. Nevertheless, the riverine flux in the Southern Ocean, although highly uncertain, is small according to a recent study (*50*) and is thus neglected in this study following the REgional Carbon Cycle Assessment and Processes Project Phase 2 (*3*).

### Gas transfer velocity derived from EC fluxes

Gas transfer velocities are derived from hourly EC CO_2_ flux observations combined with hourly air-sea CO_2_ fugacity measurements

$$K_{660}=\rho \bar{w\prime c\prime }/\left[\left(\alpha_{\text{ss}}f{\text{CO}}_{2w}-\alpha_{s}{f\text{CO}}_{2a}\right){\left(\mathrm{Sc}/660\right)}^{-0.5}\right]$$ (3)

*f*CO_2w_ and *f*CO_2a_ were measured with a showerhead equilibrator attached to the ship’s underway system (*51*) during the seven cruises in the Southern Ocean. In total, ~2500 hours of *f*CO_2_ were collected, with approximately half containing both quality-controlled EC CO_2_ flux and *f*CO_2_ observations. To reduce the relative uncertainty in the EC air-sea CO_2_ flux, minimize the relative impact of the cool skin effect, and enable an optimal analysis, the derived *K*_660_ was filtered to exclude periods when |*f*CO_2w_ − *f*CO_2a_| was less than 40 μatm.

## Supplementary Materials

### This PDF file includes:

Supplementary Text Figs. S1 to S12 Table S1 References
