# Supplementary material for: Direct observational evidence of strong CO2 uptake in the Southern Ocean
Source: Sci Adv. 2024 Jul 24;10(30):eadn5781. doi: 10.1126/sciadv.adn5781 (PMC11268400; doi:10.1126/sciadv.adn5781)
Supplement: Supplementary file 1 — Supplementary Text Figs. S1 to S12 Table S1 References [file sciadv.adn5781_sm.pdf]

Science Advances
AAAS

Supplementary Materials for

Direct observational evidence of strong CO_2 uptake in the Southern Ocean

Yuanxu Dong *et al.*

Corresponding author: Yuanxu Dong, ydong@geomar.de

Sci. Adv. **10**, eadn5781 (2024)
DOI: 10.1126/sciadv.adn5781

This PDF file includes:

Supplementary Text
Figs. S1 to S12
Table S1
References

Supplementary Text

Eddy covariance observations in the Southern Ocean

The basic information of the seven cruises with eddy covariance (EC) observations is summarized in Table S1. The seven cruises took place from 2019 to 2020 on the research vessel RRS *James Clark Ross* (JCR; JR18004, JR18005, JR19001, JR19002, and JR30001) and RRS *Discovery* (DY111, DY113). The setup of the EC systems on both ships can be found in previous research (*22*, *52*). Detailed information for these seven cruises can be found in the cruise reports available by searching the cruise name on the website of the British Oceanographic Data Centre (https://www.bodc.ac.uk/). JR30001 is a long cruise that includes several successive short cruises in the Southern Ocean. The cruise report of JR30001 is not yet available, but the EC system and the underway system are the same and have the same configuration as the systems used on other cruises on the JCR.

The three-dimensional (3D) sonic anemometer plus a motion sensor (IMU – Systron Donner MotionPak II or LPMS) were deployed on the top of the bow mast. The motion sensor is used to detect ship motions, and a motion correction is applied to the 3D wind signals to obtain the true wind velocity (*53*). All these cruises used a closed-path gas analyzer plus a dryer (to remove water vapor fluctuations) to measure the EC flux, which is recommended to make reliable EC air-sea CO_2 flux measurements (*54*–*57*). The EC systems on JCR used a Picarro G2311-f cavity ring-down spectrometer (Picarro Inc., Santa Clara, California, USA) as the gas analyzer, while the system based on Discovery used an LI-7200 (LICOR Biosciences, Lincoln, Nebraska, USA) infrared gas analyzer. A previous study (*22*) demonstrated that these setups provide reliable EC air-sea CO_2 flux observations.

In addition, underway seawater measurements (sea surface temperature, salinity, and seawater CO_2 fugacity) and atmospheric measurements (air temperature, pressure, relative humidity, and atmospheric CO_2 fugacity) were also made during all these cruises (*22*).

fCO_{2w} products

fCO_{2w} observations from four (JR18004, JR18005, JR19001, and JR19002) of seven cruises have been included in the SOCAT v2021 dataset, which might contribute to the relatively strong correlation between F_{SOCAT_sub} and F_{EC}. However, the fCO_{2w} from cruises DY113 and JR30001 are not included in the SOCAT v2021, but Fig. 3 in the main text shows that the observation-based fluxes can still reproduce the observed flux variabilities during these two cruises. In addition, the F_{SOCCOM_sub} (not using the cruise data) also reasonably reproduced the observed flux variabilities similar to the F_{SOCAT_sub}. This suggests that the agreement of the F_{SOCAT_sub} with the EC observations is not mainly due to the co-located fCO_{2w} data.

The MPI-SOMFFN flux product (*44*) has a 1° × 1°, monthly resolution, while the CarboScope flux product (*26*) has three versions with different resolutions. The one with 2° latitude × 2.5° longitude, daily resolution is primarily used. To examine if the resolution of the product impacts our results, we subsample the MPI-SOMFFN flux product and the CarboScope flux products with three resolutions (2° × 2.5°, daily; 2° × 2.5°, monthly; 1° × 1°, daily). The subsampled fluxes from these products with different resolutions are generally comparable with each other (Fig. S6). First, the refined temporal (daily) resolution substantially improves the agreement

between the subsampled flux and the EC flux. In addition, increasing the spatial resolution from 2° latitude × 2.5° longitude to 1° × 1° does not apparently improve the agreement between the subsampled flux and the EC flux (Fig. S6).

SOCCOM fCO_{2w} estimates are calculated from the float pH measurements and the LIARv2 multiple linear regression alkalinity calculation (*32*). For the SOCAT plus SOCCOM-based flux, the float fCO_{2w} estimates are first averaged in 1° by 1°, monthly bins, which are subsequently averaged with the equivalent SOCAT 1° by 1°, monthly bins. Air-sea fluxes are then calculated using the same procedure as the other MPI-SOMFFN and CarboScope products. For the SOCCOM-weighted flux, the same procedure is followed, except that south of 30°S and from 2014 onwards, only float data is used. This flux product was conceived as a test to determine how much the air-sea flux would change if weighted heavily toward the float fCO_{2w} estimates (*10*).

Gas transfer velocity

Widely used K_{660} parameterizations are either based on the global bomb-^{14}C inventory (K_{660_14C}) (e.g., *19*) or based on dual-tracer observations (K_{660_tracer}) (e.g., *58*). The global bomb-^{14}C inventory provides a single mean K_{660} value (18.2 ±3.6 cm h^{-1} or 16.5 ± 3.2 cm h^{-1} for unnormalized *K*) (28) for the global ocean over a half-century timescale, orders of magnitude longer than the timescale associated with gas exchange (minutes to hours). The K_{660_tracer} was based on a fit to observations typically collected over a few days. The short-term (e.g., hourly) gas exchange at high and low wind speeds tends to be averaged to an intermediate wind speed over a relatively long timescale (e.g., a few days). Therefore, K_{660_14C} and to a lesser extent K_{660_tracer} at low (below 5 m s^{-1}) and high wind speeds (above 13 m s^{-1}) are extrapolations from K_{660} at intermediate wind speed by assuming a quadratic K_{660}-wind speed relationship.

The variability in the EC-derived K_{660} at intermediate wind speeds (on the order of 60%, i.e., the standard deviation relative to the mean K_{660}) is substantially higher than can be explained by measurement uncertainty alone (~30%) (*22*). This suggests that processes other than wind speed (e.g., surfactants, waves) are influencing gas exchange (*52, 54, 59, 60*). The non-zero K_{660} at low wind speed is most likely influenced by chemical enhancement in air-sea CO_2 exchange (*61*), which is not included in the ^{14}C-based parameterization and cannot be observed by dual-tracer observations. Chemical enhancement is implicitly captured by EC observations and is included in EC-based K_{660} parameterizations. At high wind speeds, ocean waves and bubbles may play an important role in air-sea CO_2 exchange (*54, 59*). larger K_{660} may occur in regions with higher significant wave height (*Hs*) at the same wind speed (*21*). Our Southern Ocean observations (mostly east of South America) were not in a region with extremely high wave height because the westerly propagation of the wave energy is largely hindered by the South American landmass. The *Hs* of our observations is on the order of ~2.5 m (subsampled from the ERA5 reanalysis wave data set), while the mean *Hs* for the entire Southern Ocean is ~4 m (*62*).

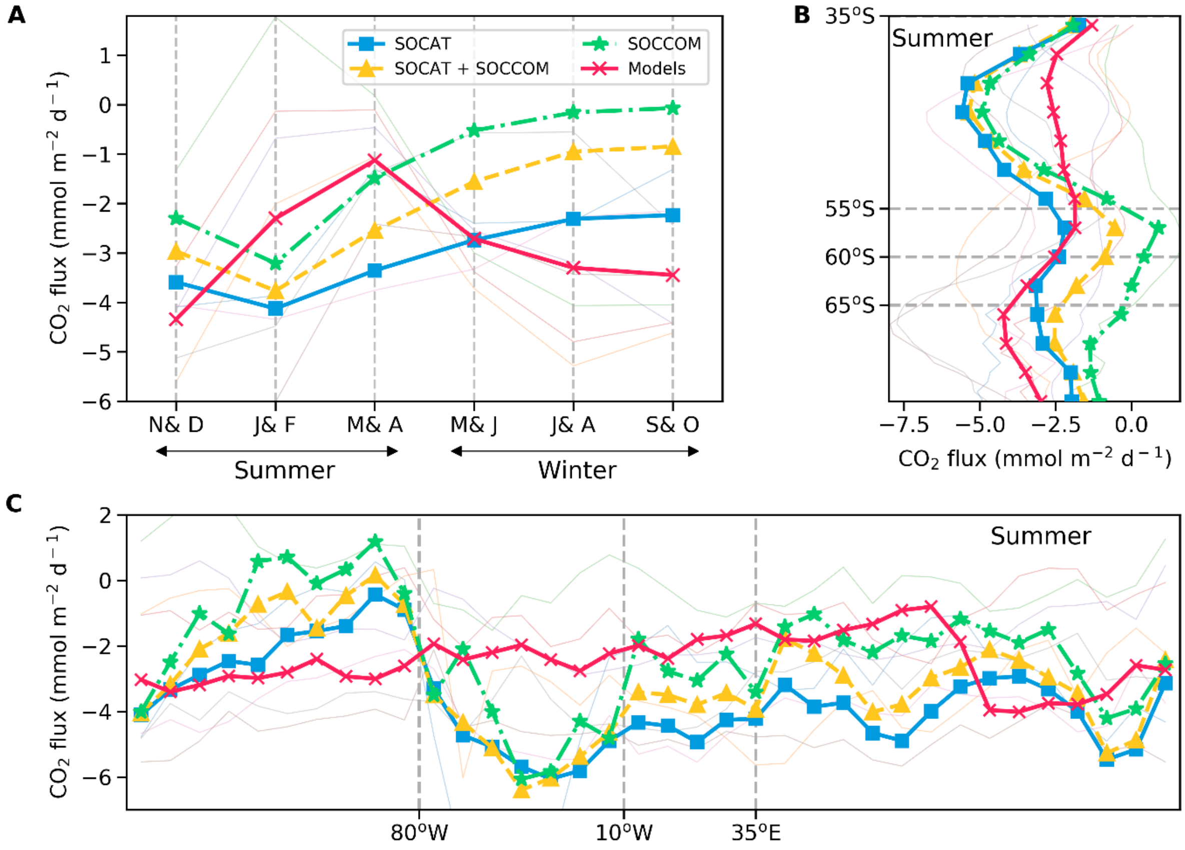

Fig. S1. Monthly, latitudinal, and longitudinal variations of the Southern Ocean CO_2 flux on average from 2015 to 2020. This figure represents the entire Southern Ocean (not subsampled). The thick blue, green, yellow, and red lines represent SOCAT (shipboard)-based, SOCCOM (float)-weighted, and SOCAT plus SOCCOM-based flux products **(**updates of the data in *10*, available from 2015 to 2020 inclusive), and the ensemble mean of eight global ocean biogeochemistry models (*23*), respectively. The eight thin lines correspond to eight individual models. (**A**) Bi-monthly averaged CO_2 flux from the observation-based products and models of the Southern Ocean. (**B**) Latitudinal variation of the CO_2 flux from observation-based products and models in the summertime Southern Ocean (November to April). (**C**) Longitudinal variation of the CO_2 flux from observation-based products and models in the summertime Southern Ocean (latitude < 35°S).

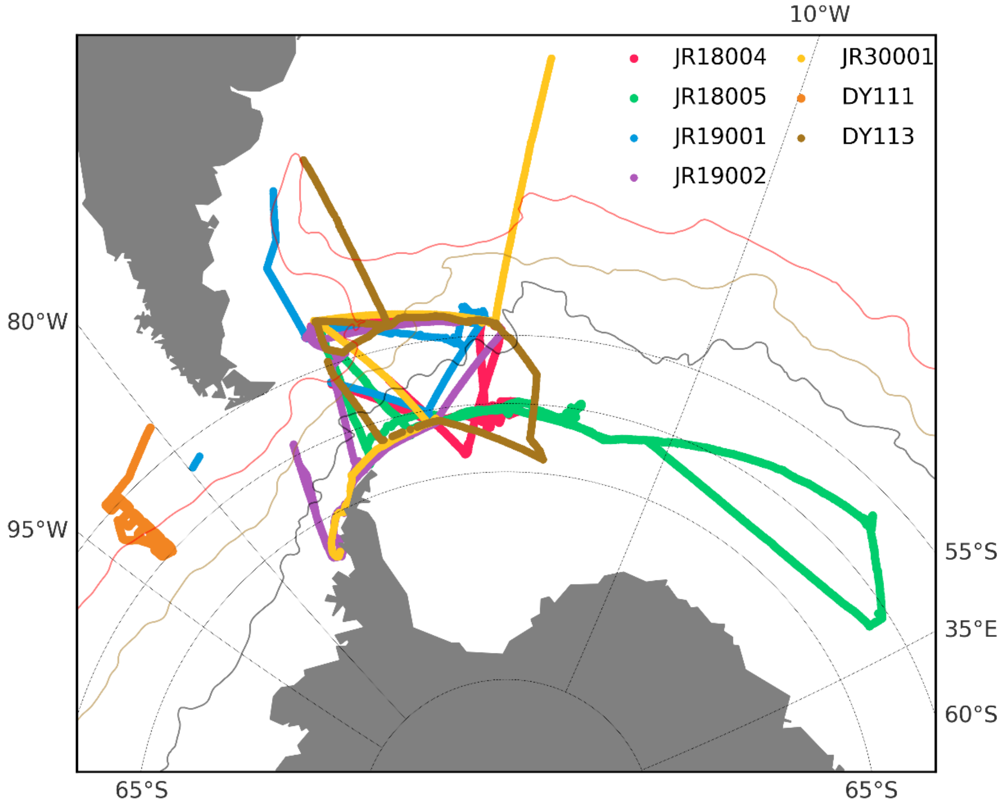

Fig. S2. Map of seven research cruises in the Southern Ocean in 2019 and 2020. Cruises JR18004, JR18005, JR19001, JR19002, and JR30001 were collected on research ship RRS *James Clark Ross*, while cruises DY111 and DY113 were collected on research vessel RRS *Discovery*. Fronts constructed from satellite altimetry data (*25*) shown as the red, brown, and black curves are for the Subantarctic Front (SAF), the Polar Front (PF), and the southern Antarctic Circumpolar Current Front (sACCF), respectively.

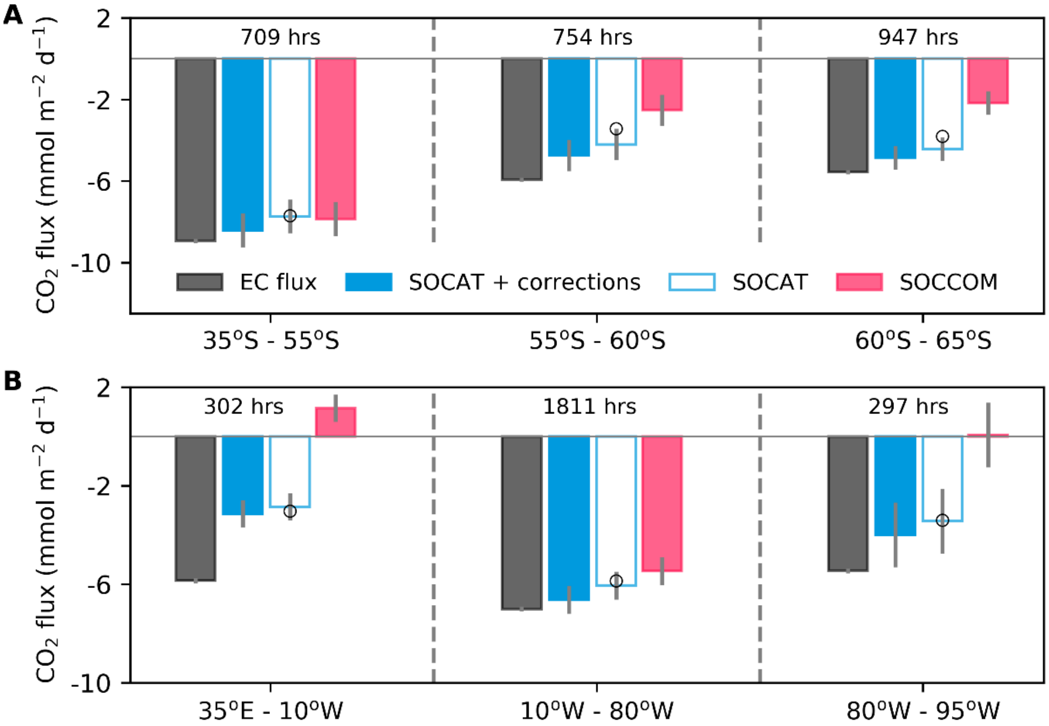

Fig. S3. Latitudinal (A) and longitudinal (B) breakdown of the EC CO_2 flux measurements and subsampled flux estimates. The four bars with different colors represent the mean of hourly EC flux measurements from the seven cruises (black), subsampled flux from SOCAT-based flux products with (filled blue) and without (unfilled blue) temperature corrections, and SOCCOM-weighted flux products (red). Open circles denote the two SOCAT-based flux products obtained using the same available interpolation methods as those for the SOCCOM-weighted products. Error bars reflecting one standard deviation provide a measure of uncertainty. Refer to the caption of Fig. 1 in the main text for the definition of the fronts SAF, PF, and sACCF.

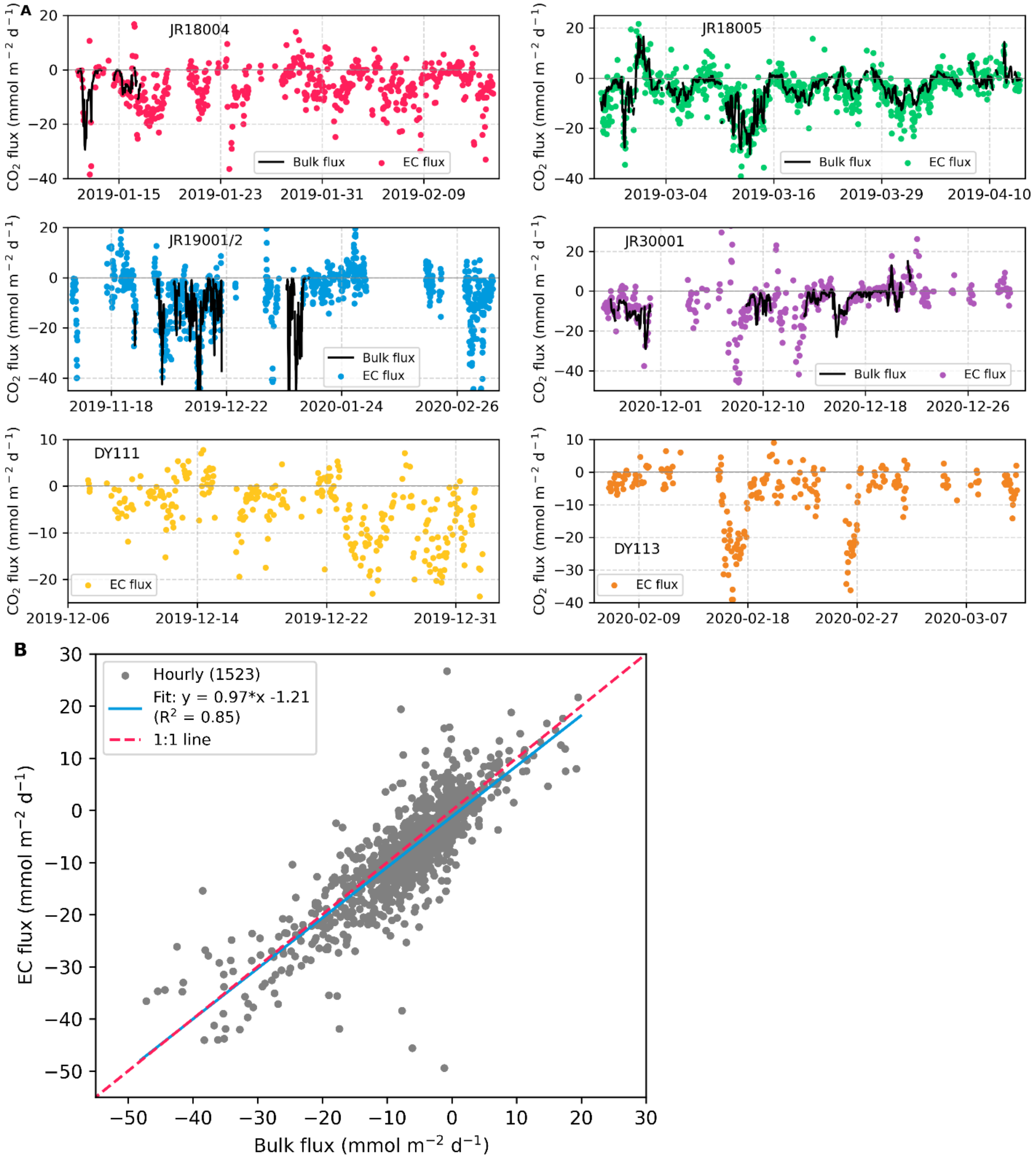

Fig. S4. Time series of hourly EC flux and bulk air-sea CO_2 flux during seven cruises in the Southern Ocean (A) and the comparison between the hourly EC flux and bulk flux (B). The bulk flux is calculated using the *in-situ* *f*CO_2 and wind speed measurements and a commonly used gas transfer velocity parameterization (*19*).

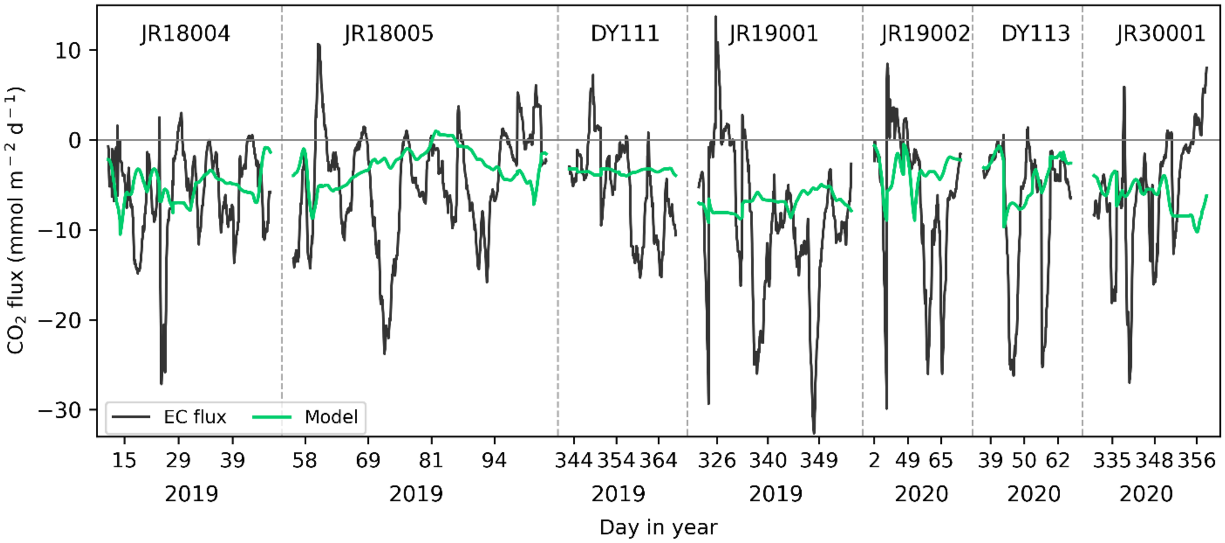

Fig. S5. Flux time series with a daily running mean. EC air-sea CO_2 flux measurements (black) from seven Southern Ocean cruises and subsampled model flux estimates (green, *23*).

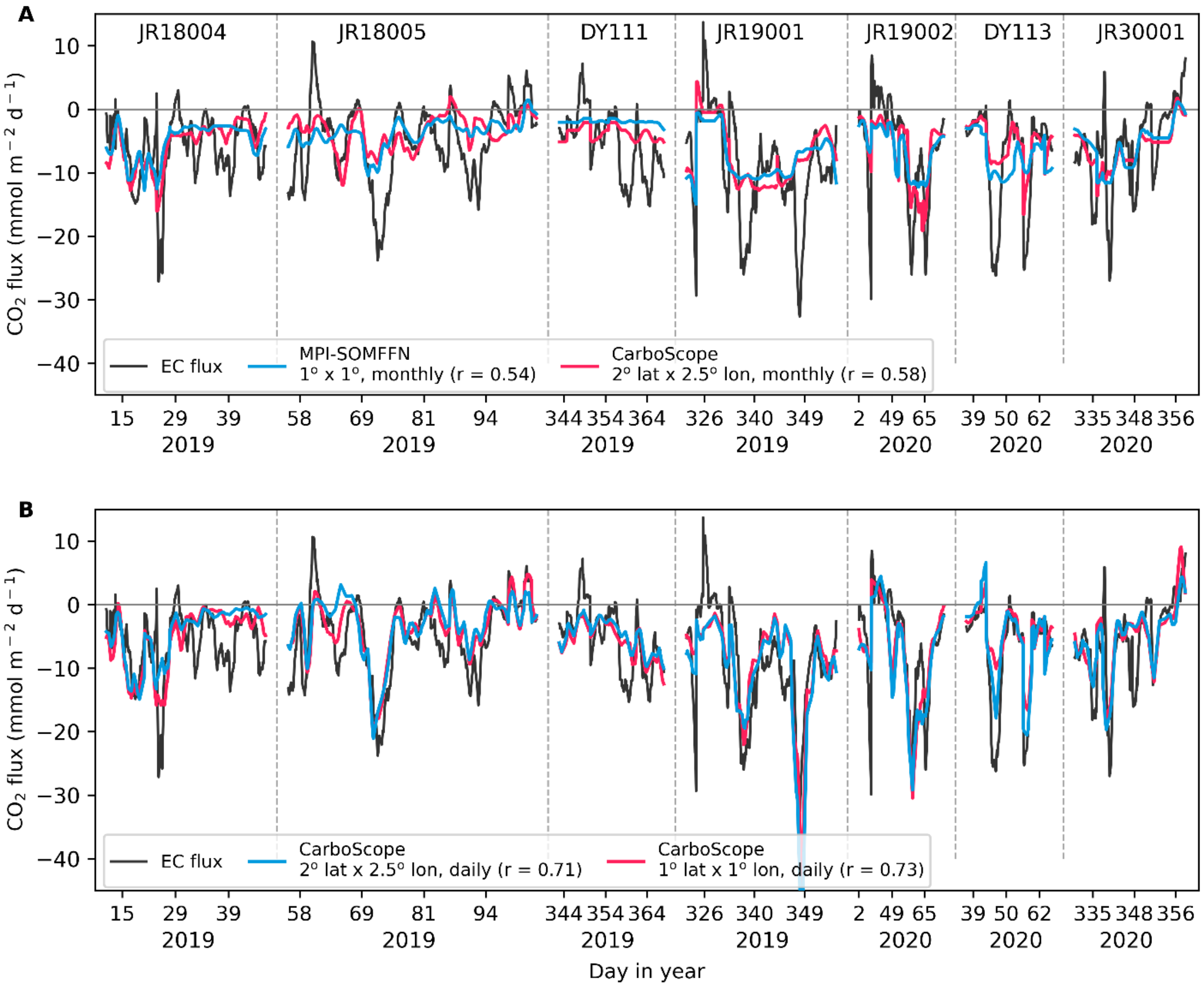

Fig. S6. Subsampled CO_2 flux according to the time and location of the EC flux measurements (black) from four SOCAT-based products: Subsampled MIP-SOMFFN flux product (*44*) with 1° × 1°, monthly resolution (blue in A); CarboScope flux product (*26*) with: 2° latitude × 2.5° longitude, monthly resolution (red in A); 2° latitude × 2.5° longitude, daily resolution (blue in B); 1°× 1°, daily resolution (red in B). The correlation coefficient (r) between the subsampled flux and the EC flux is indicated in the legend.

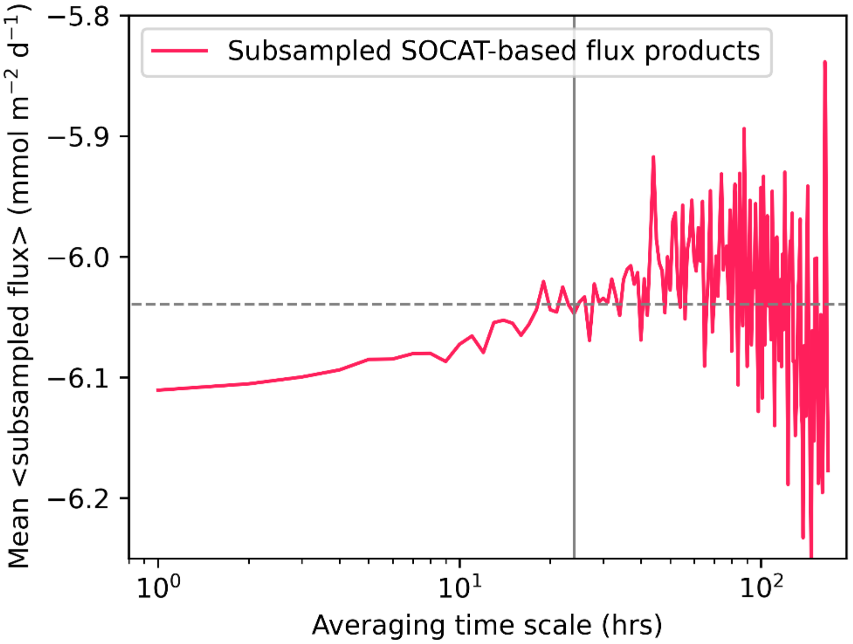

Fig. S7. The mean of the subsampled SOCAT-based CO_2 flux with different averaging time scales. The subsample is according to the time and location of each hourly EC flux. The solid-vertical line represents the 1-day time scale, and the dashed-horizontal line corresponds to the average of the mean flux with time scales between 16 and 32 hrs.

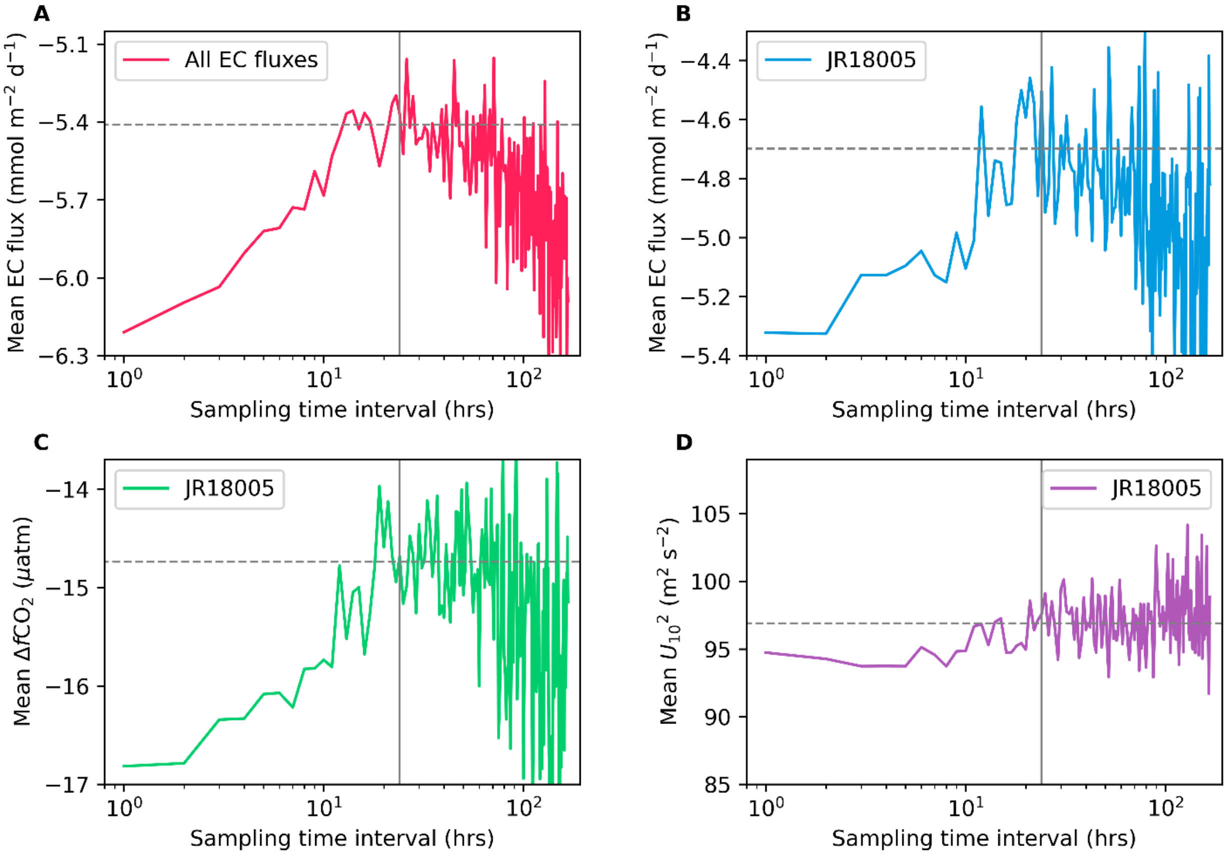

Fig. S8. The mean of the variables with different sampling time intervals. A: The mean of the entire EC flux from seven cruises. **B–D**: The mean of the EC flux (**B**), the air-sea CO_2 fugacity difference ($\Delta f CO_2$, **C**), and the square of 10-meter wind speed (U_{10}, **D**) from cruise JR18005. The solid-vertical line represents the 1-day time scale, and the dashed-horizontal line corresponds to the average of the mean flux with time scales between 16 and 32 hrs.

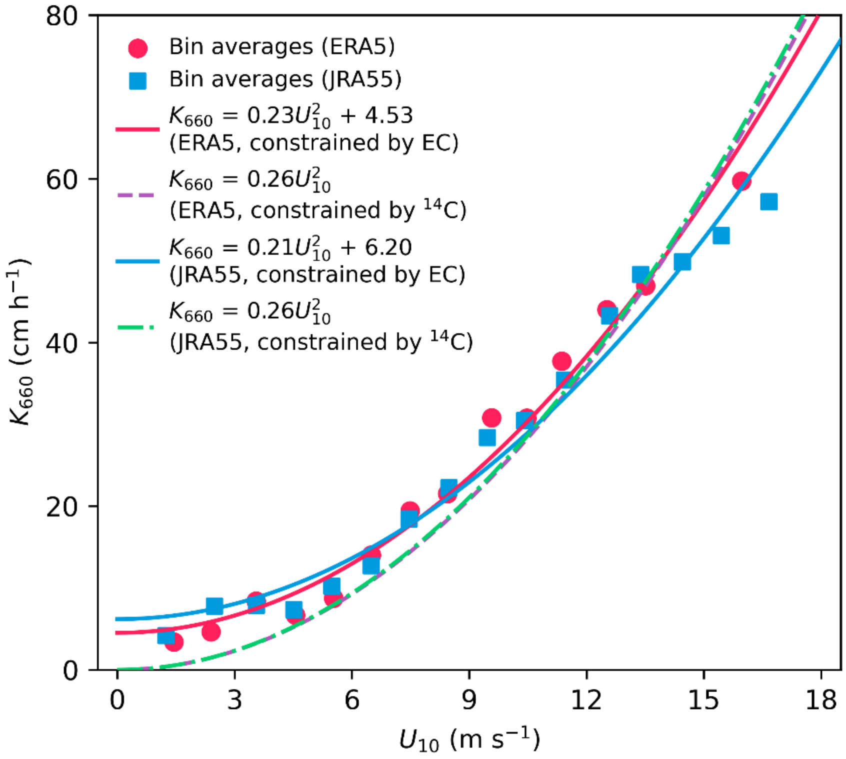

Fig. S9. Gas transfer velocities (K_{660}) versus wind speed. Red dots represent 1 m s^{-1} ERA5 wind speed (hourly resolution, *46*) bin averages of the EC-derived K_{660}, while blue squares denote 1 m s^{-1} JRA55 wind speed (3 hours resolution, *63*) bin averages of the EC-derived K_{660}. Solid curves represent the K_{660} parameterizations constrained by the EC observations (bin averages) and using the subsampled ERA5 (red) and JRA55 wind speeds (blue), respectively. Dashed lines denote the K_{660} parameterization constrained by the global ^{14}C inventory and using the global ERA5 (purple) and JRA55 wind speed product (green), respectively.

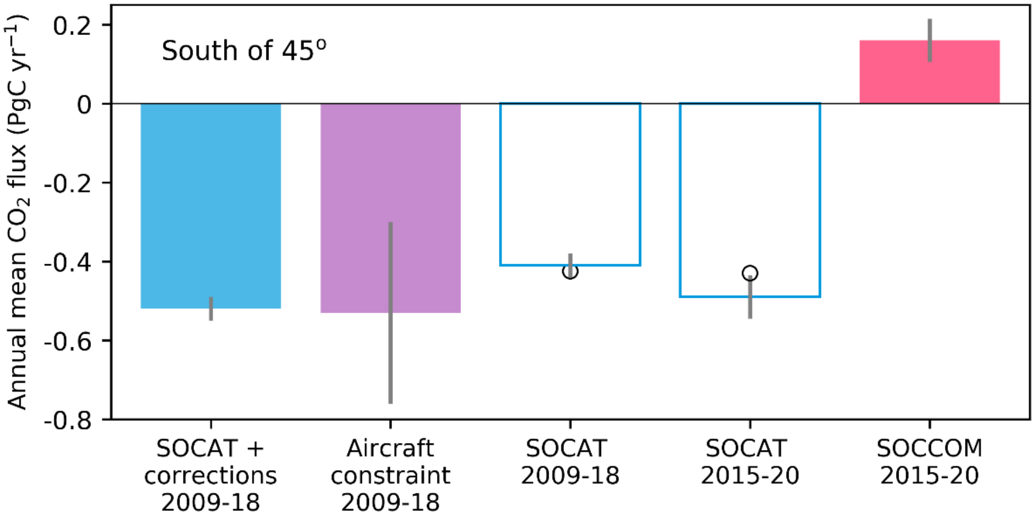

Fig. S10. Observation-based estimates of the annual mean CO_2 flux in the Southern Ocean (south of 45° here). The three bars on the left represent the fluxes averaged over 2009–2018, while the two bars on the right indicate the fluxes averaged over 2015–2020. From left to right: Ensemble mean of seven SOCAT-based flux products (*12*) with temperature corrections (filled blue) (*13*), flux constrained by the aircraft observations (purple) (29), ensemble mean of seven SOCAT-based flux products (*12*) without temperature corrections (two unfilled blue bars), ensemble mean of two SOCCOM-weighted flux products (red). Open circles denote the two SOCAT-based flux products obtained using the same available interpolation methods as those for the SOCCOM-weighted products. Error bars denote one standard deviation.

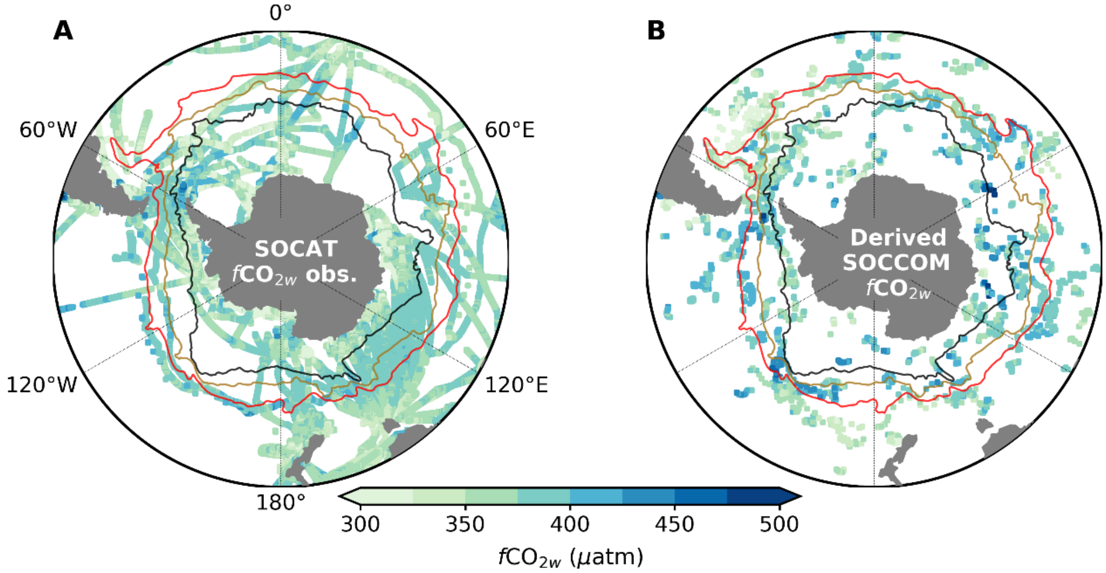

Fig. S11. SOCAT shipboard and SOCCOM float sampling distributions in the austral summer from 2015 to 2020 inclusive. Colors represent measured (**A**) and derived (**B**) seawater fCO_{2w}. Fronts constructed from satellite altimetry data (*25*) shown as the red, brown, and black curves are for the Subantarctic Front (SAF), the Polar Front (PF), and the southern Antarctic Circumpolar Current Front (sACCF), respectively.

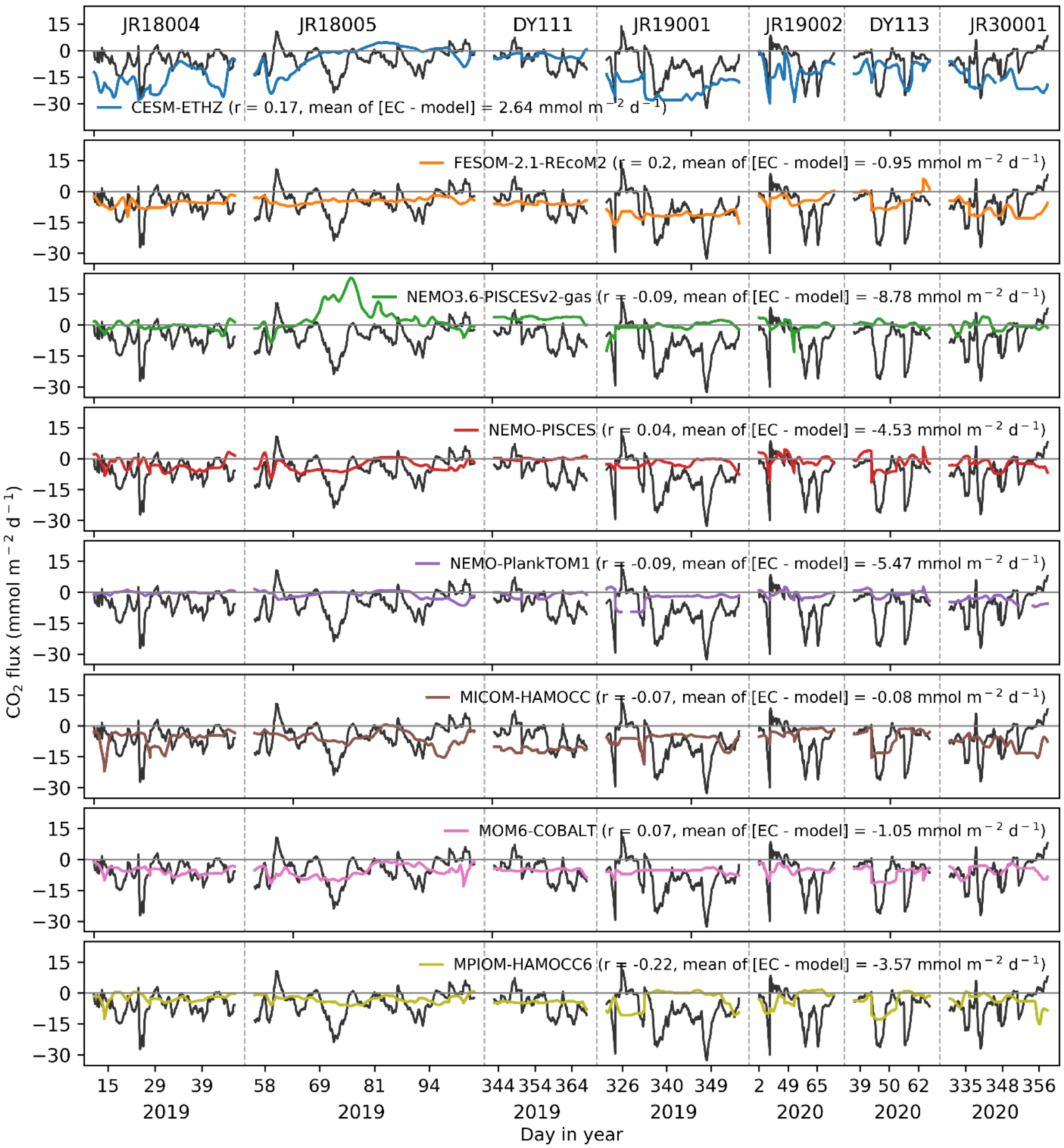

Fig. S12. EC air-sea CO_2 flux measurements and subsampled CO_2 fluxes from eight individual GOBMs with a daily running mean. The black line in each subplot represents the EC flux data, while the colored line in each subplot corresponds to the subsampled model flux. See (*23*) and references therein for details of these eight models: CESM-ETHZ (*64*), FESOM-2.1-REcoM2 (*24*), NEMO3.6-PISCESv2-gas (*65*), NEMO-PISCES (*66*), NEMO-PlankTOM12 (*67*), MICOM-HAMOCC (*68*), MOM6-COBALT (*69*), MPIOM-HAMOCC6 (*70*). The name of each model, the correlation coefficient between the subsampled model flux and the EC flux, and the mean difference between the EC flux and the subsampled model flux are indicated in the legend of each subplot.

Tab. S1. Basic information for the seven Southern Ocean cruises on which air-sea EC CO_2 fluxes were measured.

Cruise names		Platform	Sonic anemometer	Gas analyser	Date and time
JR	18004	RRS *James Clark Ross* (*JCR*)	Metek uSonic-3 Scientific + motion sensor	G2311-f + dryer	11 Jan.–15 Feb. 2019
	18005				24 Feb.–14 Apr. 2019
	19001				6 Nov.–26 Dec. 2019
	19002				27 Dec. 2019–7 Mar. 2020
	30001				1 Dec. 2020–31 Dec. 2020
DY	111	RRS *Discovery*	Gill R3-50 + motion sensor	LI-7200 + dryer	2 Dec. 2019–2 Jan. 2020
	113				5 Feb.–12 Mar. 2020

REFERENCES AND NOTES

1. S. Khatiwala, F. Primeau, T. Hall, Reconstruction of the history of anthropogenic CO_2 concentrations in the ocean. *Nature* **462**, 346–349 (2009).

2. N. Gruber, P. Landschützer, N. S. Lovenduski, The variable Southern Ocean carbon sink. *Annu. Rev. Mar. Sci.* **11**, 159–186 (2019).

3. J. Hauck, L. Gregor, C. Nissen, L. Patara, M. Hague, P. Mongwe, S. Bushinsky, S. C. Doney, N. Gruber, C. Le Quéré, M. Manizza, M. Mazloff, P. M. S. Monteiro, J. Terhaar, The Southern Ocean carbon cycle 1985–2018: Mean, seasonal cycle, trends, and storage. *Global Biogeochem. Cycles* **37**, e2023GB007848 (2023).

4. T. DeVries, C. Le Quéré, O. Andrews, S. Berthet, J. Hauck, T. Ilyina, P. Landschützer, A. Lenton, I. D. Lima, M. Nowicki, J. Schwinger, R. Séférian, Decadal trends in the ocean carbon sink. *Proc. Natl. Acad. Sci. U.S.A.* **116**, 11646–11651 (2019).

5. P. Rustogi, P. Landschützer, S. Brune, J. Baehr, The impact of seasonality on the annual air-sea carbon flux and its interannual variability. *npj Clim. Atmos. Sci.* **6**, 66 (2023).

6. D. C. E. Bakker, B. Pfeil, C. S. Landa, N. Metzl, K. M. O'Brien, A. Olsen, K. Smith, C. Cosca, S. Harasawa, S. D. Jones, S. Nakaoka, Y. Nojiri, U. Schuster, T. Steinhoff, C. Sweeney, T. Takahashi, B. Tilbrook, C. Wada, R. Wanninkhof, S. R. Alin, C. F. Balestrini, L. Barbero, N. R. Bates, A. A. Bianchi, F. Bonou, J. Boutin, Y. Bozec, E. F. Burger, W.-J. Cai, R. D. Castle, L. Chen, M. Chierici, K. Currie, W. Evans, C. Featherstone, R. A. Feely, A. Fransson, C. Goyet, N. Greenwood, L. Gregor, S. Hankin, N. J. Hardman-Mountford, J. Harlay, J. Hauck, M. Hoppema, M. P. Humphreys, C. W. Hunt, B. Huss, J. S. P. Ibánhez, T. Johannessen, R. Keeling, V. Kitidis, A. Körtzinger, A. Kozyr, E. Krasakopoulou, A. Kuwata, P. Landschützer, S. K. Lauvset, N. Lefèvre, C. Lo Monaco, A. Manke, J. T. Mathis, L. Merlivat, F. J. Millero, P. M. S. Monteiro, D. R. Munro, A. Murata, T. Newberger, A. M. Omar, T. Ono, K. Paterson, D. Pearce, D. Pierrot, L. L. Robbins, S. Saito, J. Salisbury, R. Schlitzer, B. Schneider, R. Schweitzer, R. Sieger, I. Skjelvan, K. F. Sullivan, S. C. Sutherland, A. J. Sutton, K. Tadokoro, M. Telszewski, M. Tuma, S. M. A. C. van Heuven, D. Vandemark, B. Ward, A. J. Watson, S. Xu, A multi-

decade record of high-quality fCO2 data in version 3 of the Surface Ocean CO2 Atlas (SOCAT). *Earth Syst. Sci. Data* **8**, 383–413 (2016).

7. L. Gloege, G. A. McKinley, P. Landschützer, A. R. Fay, T. L. Frölicher, J. C. Fyfe, T. Ilyina, S. Jones, N. S. Lovenduski, K. B. Rodgers, S. Schlunegger, Y. Takano, Quantifying errors in observationally based estimates of ocean carbon sink variability. *Global Biogeochem. Cycles* **35**, 1–14 (2021).

8. J. Hauck, C. Nissen, P. Landschützer, C. Rödenbeck, S. Bushinsky, A. Olsen, Sparse observations induce large biases in estimates of the global ocean CO_2 sink: An ocean model subsampling experiment. *Philos. Trans. R. Soc. A Math. Phys. Eng. Sci.* **381**, 20220063 (2023).

9. L. D. Talley, I. Rosso, I. Kamenkovich, M. R. Mazloff, J. Wang, E. Boss, A. R. Gray, K. S. Johnson, R. M. Key, S. C. Riser, Southern Ocean biogeochemical float deployment strategy, with example from the Greenwich Meridian line (GO-SHIP A12). *J. Geophys. Res. Oceans* **124**, 403–431 (2019).

10. S. M. Bushinsky, P. Landschützer, C. Rödenbeck, A. R. Gray, D. Baker, M. R. Mazloff, L. Resplandy, K. S. Johnson, J. L. Sarmiento, Reassessing Southern Ocean air-sea CO_2 flux estimates with the addition of biogeochemical float observations. *Global Biogeochem. Cycles* **33**, 1370–1388 (2019).

11. A. R. Gray, K. S. Johnson, S. M. Bushinsky, S. C. Riser, J. L. Russell, L. D. Talley, R. Wanninkhof, N. L. Williams, J. L. Sarmiento, Autonomous biogeochemical floats detect significant carbon dioxide outgassing in the high-latitude Southern Ocean. *Geophys. Res. Lett.* **45**, 9049–9057 (2018).

12. P. Friedlingstein, M. O'Sullivan, M. W. Jones, R. M. Andrew, L. Gregor, J. Hauck, C. Le Quéré, I. T. Luijkx, A. Olsen, G. P. Peters, W. Peters, J. Pongratz, C. Schwingshackl, S. Sitch, J. G. Canadell, P. Ciais, R. B. Jackson, S. R. Alin, R. Alkama, A. Arneth, V. K. Arora, N. R. Bates, M. Becker, N. Bellouin, H. C. Bittig, L. Bopp, F. Chevallier, L. P. Chini, M. Cronin, W. Evans, S. Falk, R. A. Feely, T. Gasser, M. Gehlen, T. Gkritzalis, L. Gloege, G. Grassi, N. Gruber, Ö. Gürses, I. Harris, M. Hefner, R. A. Houghton, G. C. Hurtt, Y. Iida, T. Ilyina, A. K. Jain, A. Jersild, K. Kadono, E. Kato, D. Kennedy, K. Klein Goldewijk, J. Knauer, J. I. Korsbakken, P. Landschützer, N. Lefèvre, K. Lindsay, J. Liu, Z. Liu, G. Marland, N. Mayot, M. J. McGrath, N. Metzl, N. M. Monacci, D. R. Munro, S.-I. Nakaoka, Y. Niwa, K. O'Brien, T. Ono, P. I. Palmer, N. Pan, D. Pierrot, K. Pocock, B. Poulter, L. Resplandy, E. Robertson, C. Rödenbeck, C. Rodriguez, T. M. Rosan, J. Schwinger, R. Séférian, J. D. Shutler, I. Skjelvan, T.

Steinhoff, Q. Sun, A. J. Sutton, C. Sweeney, S. Takao, T. Tanhua, P. P. Tans, X. Tian, H. Tian, B. Tilbrook, H. Tsujino, F. Tubiello, G. R. van der Werf, A. P. Walker, R. Wanninkhof, C. Whitehead, A. Willstrand Wranne, R. Wright, W. Yuan, C. Yue, X. Yue, S. Zaehle, J. Zeng, B. Zheng, Global carbon budget 2022. *Earth Syst. Sci. Data* **14**, 4811–4900 (2022).

13. Y. Dong, D. C. E. Bakker, T. G. Bell, B. Huang, P. Landschützer, P. S. Liss, M. Yang, Update on the temperature corrections of global air-sea CO_2 flux estimates. *Global Biogeochem. Cycles* **36**, e2022GB007360 (2022).

14. A. J. Watson, U. Schuster, J. D. Shutler, T. Holding, I. G. C. Ashton, P. Landschützer, D. K. Woolf, L. Goddijn-Murphy, Revised estimates of ocean-atmosphere CO_2 flux are consistent with ocean carbon inventory. *Nat. Commun.* **11**, 1–6 (2020).

15. L P. M. S. Monteiro, L. Gregor, M. Lévy, S. Maenner, C. L. Sabine, S. Swart, Intraseasonal variability linked to sampling alias in air-sea CO_2 fluxes in the Southern Ocean. *Geophys. Res. Lett.* **42**, 8507–8514 (2015).

16. A. J. Sutton, N. L. Williams, B. Tilbrook, Constraining Southern Ocean CO_2 flux uncertainty using Uncrewed Surface Vehicle observations. *Geophys. Res. Lett.* **48**, 1–9 (2021).

17. L. M. Djeutchouang, N. Chang, L. Gregor, M. Vichi, P. M. S. Monteiro, The sensitivity of pCO$_2$ reconstructions to sampling scales across a Southern Ocean sub-domain: A semi-idealized ocean sampling simulation approach. *Biogeosciences* **19**, 4171–4195 (2022).

18. S. A. Nicholson, D. B. Whitt, I. Fer, M. D. du Plessis, A. D. Lebéhot, S. Swart, A. J. Sutton, P. M. S. Monteiro, Storms drive outgassing of CO_2 in the subpolar Southern Ocean. *Nat. Commun.* **13**, 1–12 (2022).

19. R. Wanninkhof, Relationship between wind speed and gas exchange over the ocean revisited. *Limnol. Oceanogr. Methods* **12**, 351–362 (2014).

20. D. K. Woolf, J. D. Shutler, L. Goddijn-Murphy, A. J. Watson, B. Chapron, P. D. Nightingale, C. J. Donlon, J. Piskozub, M. J. Yelland, I. Ashton, T. Holding, U. Schuster, F. Girard-Ardhuin, A. Grouazel, J. F. Piolle, M. Warren, I. Wrobel-Niedzwiecka, P. E. Land, R. Torres, J. Prytherch, B. Moat, J. Hanafin,

F. Ardhuin, F. Paul, Key uncertainties in the recent air-sea flux of CO_2. *Global Biogeochem. Cycles* **33**, 1548–1563 (2019).

21. M. Yang, T. G. Bell, J. R. Bidlot, B. W. Blomquist, B. J. Butterworth, Y. Dong, C. W. Fairall, S. Landwehr, C. A. Marandino, S. D. Miller, E. S. Saltzman, A. Zavarsky, Global synthesis of air-sea CO_2 transfer velocity estimates from ship-based eddy covariance measurements. *Front. Mar. Sci.* **9**, 1–15 (2022).

22. Y. Dong, M. Yang, D. C. E. Bakker, V. Kitidis, T. G. Bell, Uncertainties in eddy covariance air-sea CO_2 flux measurements and implications for gas transfer velocity parameterisations. *Atmos. Chem. Phys.* **21**, 8089–8110 (2021).

23. P. Friedlingstein, M. W. Jones, M. O'Sullivan, R. M. Andrew, D. C. E. Bakker, J. Hauck, C. Le Quéré, G. P. Peters, W. Peters, J. Pongratz, S. Sitch, J. G. Canadell, P. Ciais, R. B. Jackson, S. R. Alin, P. Anthoni, N. R. Bates, M. Becker, N. Bellouin, L. Bopp, T. T. T. Chau, F. Chevallier, L. P. Chini, M. Cronin, K. I. Currie, B. Decharme, L. M. Djeutchouang, X. Dou, W. Evans, R. A. Feely, L. Feng, T. Gasser, D. Gilfillan, T. Gkritzalis, G. Grassi, L. Gregor, N. Gruber, Ö. Gürses, I. Harris, R. A. Houghton, G. C. Hurtt, Y. Iida, T. Ilyina, I. T. Luijkx, A. Jain, S. D. Jones, E. Kato, D. Kennedy, K. Klein Goldewijk, J. Knauer, J. I. Korsbakken, A. Körtzinger, P. Landschützer, S. K. Lauvset, N. Lefèvre, S. Lienert, J. Liu, G. Marland, P. C. McGuire, J. R. Melton, D. R. Munro, J. E. M. S. Nabel, S.-I. Nakaoka, Y. Niwa, T. Ono, D. Pierrot, B. Poulter, G. Rehder, L. Resplandy, E. Robertson, C. Rödenbeck, T. M. Rosan, J. Schwinger, C. Schwingshackl, R. Séférian, A. J. Sutton, C. Sweeney, T. Tanhua, P. P. Tans, H. Tian, B. Tilbrook, F. Tubiello, G. R. van der Werf, N. Vuichard, C. Wada, R. Wanninkhof, A. J. Watson, D. Willis, A. J. Wiltshire, W. Yuan, C. Yue, X. Yue, S. Zaehle, J. Zeng, Global carbon budget 2021. *Earth Syst. Sci. Data* **14**, 1917–2005 (2022).

24. J. Hauck, M. Zeising, C. Le Quéré, N. Gruber, D. C. E. Bakker, L. Bopp, T. T. T. Chau, Ö. Gürses, T. Ilyina, P. Landschützer, A. Lenton, L. Resplandy, C. Rödenbeck, J. Schwinger, R. Séférian, Consistency and challenges in the ocean carbon sink estimate for the global carbon budget. *Front. Mar. Sci.* **7**, 1–22 (2020).

25. Y. Park, T. Park, T. Kim, S. Lee, C. Hong, J. Lee, M. Rio, M. Pujol, M. Ballarotta, I. Durand, Observations of the Antarctic Circumpolar Current over the Udintsev Fracture Zone, the narrowest choke point in the Southern Ocean. *J. Geophys. Res. Oceans* **124**, 4511–4528 (2019).

26. C. Rödenbeck, D. C. E. Bakker, N. Metzl, A. Olsen, C. Sabine, N. Cassar, F. Reum, R. F. Keeling, M. Heimann, Interannual sea-air CO_2 flux variability from an observation-driven ocean mixed-layer scheme. *Biogeosciences* **11**, 4599–4613 (2014).

27. A. R. Fay, L. Gregor, P. Landschützer, G. A. McKinley, N. Gruber, M. Gehlen, Y. Iida, G. G. Laruelle, C. Rödenbeck, A. Roobaert, J. Zeng, SeaFlux: Harmonization of air-sea CO_2 fluxes from surface pCO_2 data products using a standardized approach. *Earth Syst. Sci. Data* **13**, 4693–4710 (2021).

28. T. Naegler, Reconciliation of excess ^{14}C-constrained global CO_2 piston velocity estimates. *Tellus B Chem. Phys. Meteorol.* **61B**, 372–384 (2009).

29. M. C. Long, B. B. Stephens, K. McKain, C. Sweeney, R. F. Keeling, E. A. Kort, E. J. Morgan, J. D. Bent, N. Chandra, F. Chevallier, Strong Southern Ocean carbon uptake evident in airborne observations. *Science* **374**, 1275–1280 (2021).

30. P. Regnier, L. Resplandy, R. G. Najjar, P. Ciais, The land-to-ocean loops of the global carbon cycle. *Nature* **603**, 401–410 (2022).

31. J. D. Müller, N. Gruber, B. R. Carter, R. A. Feely, M. Ishii, N. Lange, S. K. Lauvset, A. M. Murata, A. Olsen, F. F. Pérez, C. L. Sabine, T. Tanhua, R. Wanninkhof, D. Zhu, Decadal trends in the oceanic storage of anthropogenic carbon from 1994 to 2014. *AGU Adv.* **4**, e2023AV000875 (2023).

32. N. L. Williams, L. W. Juranek, R. A. Feely, K. S. Johnson, J. L. Sarmiento, L. D. Talley, A. G. Dickson, A. R. Gray, R. Wanninkhof, J. L. Russell, S. C. Riser, Y. Takeshita, Calculating surface ocean pCO_2 from biogeochemical Argo floats equipped with pH: An uncertainty analysis. *Global Biogeochem. Cycles* **31**, 591–604 (2017).

33. S. M. Bushinsky, I. Cerovečki, Subantarctic mode water biogeochemical formation properties and interannual variability. *AGU Adv.* **4,** e2022AV000722 (2023).

34. N. Mackay, A. Watson, J., Winter air-sea CO_2 fluxes constructed from summer observations of the polar southern ocean suggest weak outgassing. *Geophys. Res. Ocean.* **126**, e2020JC016600 (2021).

35. Y. Wu, D. C. E. Bakker, E. P. Achterberg, A. N. Silva, D. D. Pickup, X. Li, S. Hartman, D. Stappard, D. Qi, T. Tyrrell, Integrated analysis of carbon dioxide and oxygen concentrations as a quality control of ocean float data. *Commun. Earth Environ.* **3**, 92 (2022).

36. D. C. E. Bakker, S. R. Alin, N. Bates, M. Becker, R. A. Feely, T. Gkritzalis, S. D. Jones, A. Kozyr, S. K. Lauvset, N. Metzl, D. R. Munro, S. Nakaoka, Y. Nojiri, K. M. O'Brien, A. Olsen, D. Pierrot, G. Rehder, T. Steinhoff, A. J. Sutton, C. Sweeney, B. Tilbrook, C. Wada, R. Wanninkhof, J. Akl, L. Barbero, C. M. Beatty, C. F. Berghoff, H.C. Bittig, R. Bott, E. F. Burger, W. Cai, R. Castaño-Primo, J. E. Corredor, M. Cronin, E. H. De Carlo, M. D. DeGrandpre, C. Dietrich, W. M. Drennan, S. R. Emerson, I. C. Enochs, K. Enyo, L. Epherra, W. Evans, B. Fiedler, M. Fontela, C. Frangoulis, M. Gehrung, L. Giannoudi, M. Glockzin, B. Hales, S. D. Howden, J. S.P. Ibánhez, L. Kamb, A. Körtzinger, N. Lefèvre, C. Lo Monaco, V. A. Lutz, V. A. Macovei, S. Maenner Jones, D. Manalang, D. P. Manzello, N. Metzl, J. Mickett, F. J. Millero, N. M. Monacci, J. M. Morell, S. Musielewicz, C. Neill, T. Newberger, J. Newton, S. Noakes, S. R. Ólafsdóttir, T. Ono, J. Osborne, X. A. Padín, M. Paulsen, L. Perivoliotis, W. Petersen, G. Petihakis, A. J. Plueddemann, C. Rodriguez, A. Rutgersson, C. L. Sabine, J. E. Salisbury, R. Schlitzer, I. Skjelvan, N. Stamataki, K. F. Sullivan, S. C. Sutherland, M. T'Jampens, K. Tadokoro, T. Tanhua, M. Telszewski, H. Theetaert, M. Tomlinson, D. Vandemark, A. Velo, Y. G. Voynova, R. A. Weller, C. Whitehead, C. Wimart-Rousseau, Surface Ocean CO2 Atlas Database Version 2023 (SOCATv2023) (NCEI Accession 0278913). NOAA National Centers for Environmental Information Dataset. https://doi.org/10.25921/r7xa-bt92.

37. R. Wanninkhof, W. E. Asher, D. T. Ho, C. Sweeney, W. R. McGillis, Advances in quantifying air-sea gas exchange and environmental forcing. *Annu. Rev. Mar. Sci.* **1**, 213–244 (2009).

38. R. F. Weiss, Carbon dioxide in water and seawater: The solubility of a non-ideal gas. *Mar. Chem.* **2**, 203–215 (1974).

39. D. K. Woolf, P. E. Land, J. D. Shutler, L. M. Goddijn-Murphy, C. J. Donlon, On the calculation of air-sea fluxes of CO_2 in the presence of temperature and salinity gradients. *J. Geophys. Res. Oceans* **121**, 1229–1248 (2016).

40. T. T. T. Chau, M. Gehlen, F. Chevallier, A seamless ensemble-based reconstruction of surface ocean *p*CO_2 and air-sea CO_2 fluxes over the global coastal and open oceans. *Biogeosciences* **19**, 1087–1109 (2022).

41. L. Gregor, N. Gruber, OceanSODA-ETHZ: A global gridded data set of the surface ocean carbonate system for seasonal to decadal studies of ocean acidification. *Earth Syst. Sci. Data* **13**, 777–808 (2021).

42. L. Gregor, A. D. Lebehot, S. Kok, P. M. S. Monteiro, A comparative assessment of the uncertainties of global surface ocean CO_2 estimates using a machine-learning ensemble (CSIR-ML6 version 2019a) – have we hit the wall? *Geosci. Model Dev.* **12**, 5113–5136 (2019).

43. Y. Iida, Y. Takatani, A. Kojima, M. Ishii, Global trends of ocean CO_2 sink and ocean acidification: An observation-based reconstruction of surface ocean inorganic carbon variables. *J. Oceanogr.* **77**, 323–358 (2021).

44. P. Landschützer, N. Gruber, D. C. E. Bakker, Decadal variations and trends of the global ocean carbon sink. *Global Biogeochem. Cycles* **30**, 1396–1417 (2016).

45. J. Zeng, Y. Nojiri, P. Landschützer, M. Telszewski, S. Nakaoka, A global surface ocean *f*CO_2 climatology based on a feed-forward neural network. *J. Atmos. Ocean. Technol.* **31**, 1838–1849 (2014).

46. H. Hersbach, B. Bell, P. Berrisford, S. Hirahara, A. Horányi, J. Muñoz-Sabater, J. Nicolas, C. Peubey, R. Radu, D. Schepers, A. Simmons, C. Soci, S. Abdalla, X. Abellan, G. Balsamo, P. Bechtold, G. Biavati, J. Bidlot, M. Bonavita, G. De Chiara, P. Dahlgren, D. Dee, M. Diamantakis, R. Dragani, J. Flemming, R. Forbes, M. Fuentes, A. Geer, L. Haimberger, S. Healy, R. J. Hogan, E. Hólm, M. Janisková, S. Keeley, P. Laloyaux, P. Lopez, C. Lupu, G. Radnoti, P. de Rosnay, I. Rozum, F. Vamborg, S. Villaume, J. N. Thépaut, The ERA5 global reanalysis. *Q. J. R. Meteorol. Soc.* **146**, 1999–2049 (2020).

47. R. W. Reynolds, T. M. Smith, C. Liu, D. B. Chelton, K. S. Casey, M. G. Schlax, Daily high-resolution-blended analyses for sea surface temperature. *J. Climate* **20**, 5473–5496 (2007).

48. C. W. Fairall, E. F. Bradley, J. S. Godfrey, G. A. Wick, J. B. Edson, G. S. Young, Cool-skin and warm-layer effects on sea surface temperature. *J. Geophys. Res. Oceans* **101**, 1295–1308 (1996).

49. F. Xu, A. Ignatov, In situ SST quality monitor (i Quam). *J. Atmos. Ocean. Technol.* **31**, 164–180 (2014).

50. F. Lacroix, T. Ilyina, J. Hartmann, Oceanic CO_2 outgassing and biological production hotspots induced by pre-industrial river loads of nutrients and carbon in a global modeling approach. *Biogeosciences* **17**, 55–88 (2020).

51. V. Kitidis, I. Brown, N. Hardman-Mountford, N. Lefèvre, Surface ocean carbon dioxide during the Atlantic Meridional Transect (1995–2013); evidence of ocean acidification. *Prog. Oceanogr.* **158**, 65–75 (2017).

52. M. Yang, T. J. Smyth, V. Kitidis, I. J. Brown, C. Wohl, M. J. Yelland, T. G. Bell, Natural variability in air-sea gas transfer efficiency of CO_2. *Sci. Rep.* **11**, 1–9 (2021).

53. J. B. Edson, A. A. Hinton, K. E. Prada, J. E. Hare, C. W. Fairall, Direct covariance flux estimates from mobile platforms at sea. *J. Atmos. Ocean. Technol.* **15**, 547–562 (1998).

54. B. W. Blomquist, S. E. Brumer, C. W. Fairall, B. J. Huebert, C. J. Zappa, I. M. Brooks, M. Yang, L. Bariteau, J. Prytherch, J. E. Hare, H. Czerski, A. Matei, R. W. Pascal, Wind speed and sea state dependencies of air-sea gas transfer: Results from the High Wind Speed Gas Exchange Study (HiWinGS). *J. Geophys. Res. Oceans* **122**, 8034–8062 (2017).

55. S. Landwehr, S. D. Miller, M. J. Smith, E. S. Saltzman, B. Ward, Analysis of the PKT correction for direct CO_2 flux measurements over the ocean. *Atmos. Chem. Phys.* **14**, 3361–3372 (2014).

56. S. D. Miller, C. Marandino, E. S. Saltzman, Ship-based measurement of air-sea CO_2 exchange by eddy covariance. *J. Geophys. Res. Atmos.* **115**, 1–14 (2010).

57. E. Nilsson, H. Bergström, A. Rutgersson, E. Podgrajsek, M. B. Wallin, G. Bergström, E. Dellwik, S. Landwehr, B. Ward, Evaluating humidity and sea salt disturbances on CO_2 flux measurements. *J. Atmos. Ocean. Technol.* **35**, 859–875 (2018).

58. D. T. Ho, C. S. Law, M. J. Smith, P. Schlosser, M. Harvey, P. Hill, Measurements of air-sea gas exchange at high wind speeds in the Southern Ocean: Implications for global parameterizations. *Geophys. Res. Lett.* **33**, L16611 (2006).

59. T. G. Bell, S. Landwehr, S. D. Miller, W. J. De Bruyn, A. H. Callaghan, B. Scanlon, B. Ward, M. Yang, E. S. Saltzman, Estimation of bubble-mediated air-sea gas exchange from concurrent DMS and CO_2 transfer velocities at intermediate-high wind speeds. *Atmos. Chem. Phys.* **17**, 9019–9033 (2017).

60. R. Pereira, I. Ashton, B. Sabbaghzadeh, J. D. Shutler, R. C. Upstill-Goddard, Reduced air-sea CO_2 exchange in the Atlantic Ocean due to biological surfactants. *Nat. Geosci.* **11**, 492–496 (2018).

61. C. W. Fairall, M. Yang, S. E. Brumer, B. W. Blomquist, J. B. Edson, C. J. Zappa, L. Bariteau, S. Pezoa, T. G. Bell, E. S. Saltzman, Air-sea trace gas fluxes: Direct and indirect measurements. *Front. Mar. Sci.* **9**, 1–16 (2022).

62. I. R. Young, E. Fontaine, Q. Liu, A. V Babanin, The wave climate of the Southern Ocean. *J. Phys. Oceanogr.* **50**, 1417–1433 (2020).

63. S. Kobayashi, Y. Ota, Y. Harada, A. Ebita, M. Moriya, H. Onoda, K. Onogi, H. Kamahori, C. Kobayashi, H. Endo, The JRA-55 reanalysis: General specifications and basic characteristics. *J. Meteorol. Soc. Jpn. Ser.* **93**, 5–48 (2015).

64. S. C. Doney, I. Lima, R. A. Feely, D. M. Glover, K. Lindsay, N. Mahowald, J. K. Moore, R. Wanninkhof, Mechanisms governing interannual variability in upper-ocean inorganic carbon system and air-sea CO_2 fluxes: Physical climate and atmospheric dust. *Deep Sea Res. Part II Top. Stud. Oceanogr.* **56**, 640–655 (2009).

65. S. Berthet, R. Séférian, C. Bricaud, M. Chevallier, A. Voldoire, C. Ethé, Evaluation of an online grid-coarsening algorithm in a global Eddy-Admitting ocean biogeochemical model. *J. Adv. Model. Earth Syst.* **11**, 1759–1783 (2019).

66. O. Aumont, C. Ethé, A. Tagliabue, L. Bopp, M. Gehlen, PISCES-v2: An ocean biogeochemical model for carbon and ecosystem studies. *Geosci. Model Dev.* **8**, 2465–2513 (2015).

67. E. T. Buitenhuis, T. Hashioka, C. Le Quéré, Combined constraints on global ocean primary production using observations and models. *Global Biogeochem. Cycles* **27**, 847–858 (2013).

68. J. Schwinger, N. Goris, J. F. Tjiputra, I. Kriest, M. Bentsen, I. Bethke, M. Ilicak, K. M. Assmann, C. Heinze, Evaluation of NorESM-OC (versions 1 and 1.2), the ocean carbon-cycle stand-alone configuration of the Norwegian Earth System Model (NorESM1). *Geosci. Model Dev.* **9**, 2589–2622 (2016).

69. A. Adcroft, W. Anderson, V. Balaji, C. Blanton, M. Bushuk, C. O. Dufour, J. P. Dunne, S. M. Griffies, R. Hallberg, M. J. Harrison, The GFDL global ocean and sea ice model OM4. 0: Model description and simulation features. *J. Adv. Model. Earth Syst.* **11**, 3167–3211 (2019).

70. H. Paulsen, T. Ilyina, K. D. Six, I. Stemmler, Incorporating a prognostic representation of marine nitrogen fixers into the global ocean biogeochemical model HAMOCC. *J. Adv. Model. Earth Syst.* **9**, 438–464 (2017).
